# DNAm-based signatures of accelerated aging and mortality in blood are associated with low renal function

**DOI:** 10.1186/s13148-021-01082-w

**Published:** 2021-06-02

**Authors:** Pamela R. Matías-García, Cavin K. Ward-Caviness, Laura M. Raffield, Xu Gao, Yan Zhang, Rory Wilson, Xīn Gào, Jana Nano, Andrew Bostom, Elena Colicino, Adolfo Correa, Brent Coull, Charles Eaton, Lifang Hou, Allan C. Just, Sonja Kunze, Leslie Lange, Ethan Lange, Xihong Lin, Simin Liu, Jamaji C. Nwanaji-Enwerem, Alex Reiner, Jincheng Shen, Ben Schöttker, Pantel Vokonas, Yinan Zheng, Bessie Young, Joel Schwartz, Steve Horvath, Ake Lu, Eric A. Whitsel, Wolfgang Koenig, Jerzy Adamski, Juliane Winkelmann, Hermann Brenner, Andrea A. Baccarelli, Christian Gieger, Annette Peters, Nora Franceschini, Melanie Waldenberger

**Affiliations:** 1 TUM School of Medicine, Technical University of Munich, Munich, Germany; 2 Research Unit Molecular Epidemiology, Institute of Epidemiology, Helmholtz Zentrum München, German Research Center for Environmental Health, Munich/Neuherberg, Germany; 3 Institute of Epidemiology, Helmholtz Zentrum München, German Research Center for Environmental Health, Munich/Neuherberg, Germany; 4 German Center for Cardiovascular Research (DZHK), Partner Site Munich Heart Alliance, Munich, Germany; 5 Center for Public Health and Environmental Assessment, US Environmental Protection Agency, Chapel Hill, NC USA; 6 Department of Genetics, University of North Carolina, Chapel Hill, NC USA; 7 Laboratory of Precision Environmental Health, Mailman School of Public Health, Columbia University, New York, NY USA; 8 Division of Clinical Epidemiology and Aging Research, German Cancer Research Center (DKFZ), Heidelberg, Germany; 9 German Cancer Consortium (DKTK), German Cancer Research Center (DKFZ), Heidelberg, Germany; 10 German Center for Diabetes Research (DZD), Neuherberg, Germany; 11 Center For Primary Care and Prevention, Memorial Hospital of Rhode Island, Pawtucket, RI USA; 12 Department of Environmental Medicine and Public Health, Icahn School of Medicine at Mount Sinai, New York, NY USA; 13 Departments of Medicine and Pediatrics, University of Mississippi Medical Center, Jackson, MS USA; 14 Department of Biostatistics, Harvard T.H. Chan School of Public Health, Boston, MA USA; 15 Department of Family Medicine, Warren Alpert Medical School, Brown University, Providence, RI USA; 16 Department of Preventive Medicine, Feinberg School of Medicine, Northwestern University, Chicago, IL USA; 17 Department of Medicine, University of Colorado Denver, Anschutz Medical Campus, Aurora, CO USA; 18 Veterans Affairs Normative Aging Study, Veterans Affairs Boston Healthcare System, Department of Medicine, Boston University School of Medicine, Boston, MA USA; 19 Department of Epidemiology, School of Public Health, Brown University, Providence, RI USA; 20 Department of Environmental Health, Harvard T.H. Chan School of Public Health, Boston, MA USA; 21 Department of Epidemiology, University of Washington, Seattle, WA USA; 22 Department of Population Health Sciences, School of Medicine, University of Utah, Salt Lake City, UT USA; 23 Network Aging Research, University of Heidelberg, Heidelberg, Germany; 24 Nephrology, Hospital and Specialty Medicine and Center for Innovation for Veteran-Centered and Value Driven Care, Veterans Affairs Puget Sound Health Care System, Seattle, WA USA; 25 Division of Nephrology, Kidney Research Institute, University of Washington, Seattle, WA USA; 26 Department of Human Genetics, David Geffen School of Medicine, University of California Los Angeles, Los Angeles, CA USA; 27 Department of Epidemiology, Gillings School of Global Public Health, University of North Carolina, Chapel Hill, NC USA; 28 Department of Medicine, School of Medicine, University of North Carolina, Chapel Hill, NC USA; 29 Deutsches Herzzentrum München, Technische Universität München, Munich, Germany; 30 Institute of Epidemiology and Medical Biometry, University of Ulm, Ulm, Germany; 31 Research Unit Molecular Endocrinology and Metabolism, Genome Analysis Center, Helmholtz Zentrum München, German Research Center for Environmental Health, Munich/Neuherberg, Germany; 32 Chair for Experimental Genetics, Technical University of Munich, Freising-Weihenstephan, Germany; 33 Department of Biochemistry, Yong Loo Lin School of Medicine, National University of Singapore, Singapore, Singapore; 34 Institute of Neurogenomics, Helmholtz Zentrum München, German Research Center for Environmental Health, Munich/Neuherberg, Germany; 35 Chair Neurogenetics, Klinikum rechts der Isar, Technical University of Munich, Munich, Germany; 36 Institute of Human Genetics, Klinikum rechts der Isar, Technical University of Munich, Munich, Germany; 37 Munich Cluster for Systems Neurology (SyNergy), Munich, Germany

**Keywords:** Aging, Kidney function, Epigenetic age acceleration, DNAm age, Glomerular filtration rate, UACR, Serum urate

## Abstract

**Background:**

The difference between an individual's chronological and DNA methylation predicted age (DNAmAge), termed DNAmAge acceleration (DNAmAA), can capture life-long environmental exposures and age-related physiological changes reflected in methylation status. Several studies have linked DNAmAA to morbidity and mortality, yet its relationship with kidney function has not been assessed. We evaluated the associations between seven DNAm aging and lifespan predictors (as well as GrimAge components) and five kidney traits (estimated glomerular filtration rate [eGFR], urine albumin-to-creatinine ratio [uACR], serum urate, microalbuminuria and chronic kidney disease [CKD]) in up to 9688 European, African American and Hispanic/Latino individuals from seven population-based studies.

**Results:**

We identified 23 significant associations in our large trans-ethnic meta-analysis (*p* < 1.43E−03 and consistent direction of effect across studies). Age acceleration measured by the Extrinsic and PhenoAge estimators, as well as Zhang’s 10-CpG epigenetic mortality risk score (MRS), were associated with all parameters of poor kidney health (lower eGFR, prevalent CKD, higher uACR, microalbuminuria and higher serum urate). Six of these associations were independently observed in European and African American populations. MRS in particular was consistently associated with eGFR (*β* =  − 0.12, 95% CI = [− 0.16, − 0.08] change in log-transformed eGFR per unit increase in MRS, *p* = 4.39E−08), prevalent CKD (odds ratio (OR) = 1.78 [1.47, 2.16], p = 2.71E-09) and higher serum urate levels (*β* = 0.12 [0.07, 0.16], *p* = 2.08E−06). The “first-generation” clocks (Hannum, Horvath) and GrimAge showed different patterns of association with the kidney traits. Three of the DNAm-estimated components of GrimAge, namely adrenomedullin, plasminogen-activation inhibition 1 and pack years, were positively associated with higher uACR, serum urate and microalbuminuria.

**Conclusion:**

DNAmAge acceleration and DNAm mortality predictors estimated in whole blood were associated with multiple kidney traits, including eGFR and CKD, in this multi-ethnic study. Epigenetic biomarkers which reflect the systemic effects of age-related mechanisms such as immunosenescence, inflammaging and oxidative stress may have important mechanistic or prognostic roles in kidney disease. Our study highlights new findings linking kidney disease to biological aging, and opportunities warranting future investigation into DNA methylation biomarkers for prognostic or risk stratification in kidney disease.

**Supplementary Information:**

The online version contains supplementary material available at 10.1186/s13148-021-01082-w.

## Background

The kidneys are responsible for maintenance of homeostasis and blood filtration, and their function is most commonly clinically assessed by measuring serum creatinine levels to estimate glomerular filtration rate (eGFR) [1, 2]. Chronic kidney disease (CKD), defined by low eGFR (< 60 ml/min/m^2^) and/or presence of protein in urine, is an increasingly prevalent non-communicable disease with a considerable burden worldwide [3–5]. Increased urinary albumin-to-creatinine ratio (uACR) is a marker of kidney injury, measured to identify early kidney damage which can precede eGFR decline (for example, diabetic nephropathy) [6]. Albuminuria is a predictor of CKD progression and mortality [6, 7], whereas high serum levels of urate, a molecule of purine nucleotide metabolism excreted by the kidney, is a risk factor for incident cardiovascular and kidney disease, and also a biomarker of low eGFR [8].

DNA methylation (DNAm), defined as the covalent addition of a methyl group to a DNA nucleotide (usually the cytosine of a cytosine-guanine dinucleotide [CpG]), is the most extensively studied epigenetic mechanism, and its role in numerous conditions and diseases has been demonstrated [9]. Age-predicting algorithms based on the percentages of DNAm observed at sets of CpGs, such as the ones proposed by Hannum [10] and Horvath [11], have been used to predict an individual’s age (DNAmAge) and assess biological aging by calculating the difference between an individual’s predicted and chronological age—a concept known as DNAmAge acceleration (DNAmAA) [12, 13]. Other measures have been derived to assess specific aspects of aging mechanisms, such as intrinsic epigenetic age acceleration (IEAA), which assesses aging independent of blood immune system changes [14], or extrinsic epigenetic age acceleration (EEAA) [15, 16], which specifically estimates aging as related to the immune system and reflected in changes in blood immune cell-type proportions. A “second generation” of DNAm-based aging signatures incorporated physiological markers to better capture changes in traditional biological aging biomarkers [12]. PhenoAge was developed as a marker meant to mirror physiological dysregulation as reflected in changes in age and 9 additional age-related features, such as C-reactive protein and serum glucose [17]. GrimAge is a mortality predictor based on mortality-related DNAm-estimated traits [18]. Another DNAm-based lifespan predictor, the 10-CpG epigenetic mortality risk score (MRS), stands out for its simplicity and its recent validation [19, 20].

Multiple studies have shown, although with varying findings, a positive relationship between DNAmAge measured in blood and aging-related diseases and mortality [12, 13, 21]. DNAmAge is associated with all-cause mortality [22, 23], frailty [24], cognitive function and physical fitness [25], body mass index (BMI) [26] and obesity [27], lifetime stress [28] and a number of other age-related conditions [12]. The available evidence points to DNAmAge as a potential global biomarker of biological aging and health, though potential for publication bias must be considered [21]. Although some of the “second-generation” DNAm-based aging measures include proteins or markers known to be associated with kidney function [17, 18], whether these and other DNAm-based predictors are correlated with different parameters of kidney aging and low function has not been investigated [13, 29, 30].

We evaluated the association between five kidney traits (eGFR, prevalent CKD, uACR, microalbuminuria and serum urate) and seven DNAm-based age and/or lifespan predictors (HannumAA, HorvathAA, EEAA, IEAA, PhenoAA, GrimAA and MRS) in up to seven population-based studies in a large trans-ethnic meta-analysis. We also evaluated kidney trait associations with secondary DNAm-based predictors: categorical epigenetic mortality risk score (MRS) variables, and eight DNAm-estimated traits underlying the GrimAge mortality predictor. We additionally performed ethnic-specific meta-analyses to identify robust associations across cohorts of different ethnicities.

## Results

### Study characteristics

**Fig. 1:**
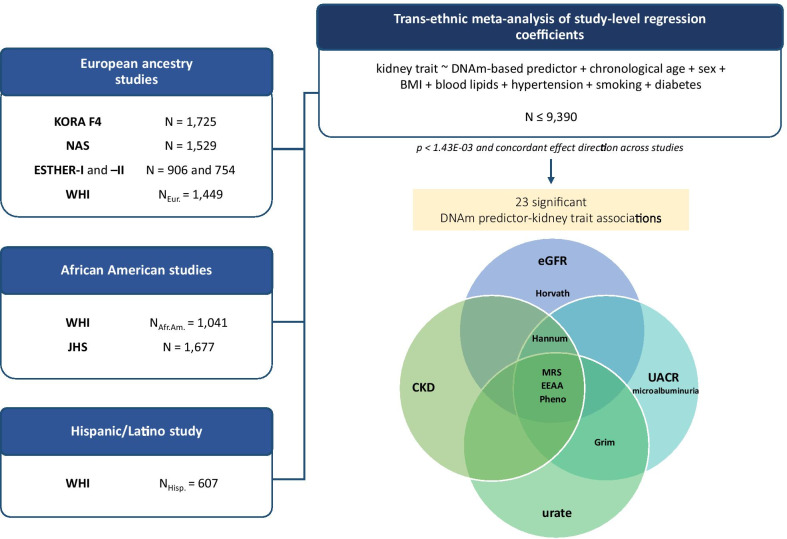
Study design. Regression analyses were conducted in each study by modeling DNAm-based predictors as independent variables and kidney traits as dependent variables, adjusting for confounders. Results from the fully adjusted model (with chronological age, sex, BMI, log-transformed triglycerides, HDL, hypertension, smoking status, diabetes as covariates and baseline eGFR for serum urate analyses) were meta-analyzed using inverse-variance weighted fixed-effects and random-effects models. We based our main interpretations on the fixed-effects results; if heterogeneity was large (*I*^2^ > 0.50 and Cochran’s *Q p*_het_ < 0.05), we based our interpretations on the random-effects results. The Venn diagram shows the set of 23 statistically significant associations between DNAm-based predictors and kidney traits identified in the trans-ethnic meta-analysis (*p* < 1.43E−03 and consistent direction of effect across studies)

We conducted a trans-ethnic meta-analysis of seven DNAm-based age/lifespan predictors and five kidney traits using data from seven population-based cohorts (Fig. 1, population characteristics in Table 1). Serum creatinine-based traits (eGFR and prevalent CKD) were available for all European ancestry (*k* = 5), African American (*k* = 2) and one additional Hispanic/Latino study (*N* = 9688). Sample sizes for the other traits were smaller: serum urate was available in four studies with European ancestry and one study with African American participants (*N* = 5903), while uACR and microalbuminuria were available in three of the European ancestry studies and one African American study (*N* = 4110). Additional details on the cohorts are provided in Additional file 1: Table S1.

**Table 1:** Population characteristics

Traits	KORA	ESTHER-I	ESTHER-II	NAS	WHI			JHS
Ethnic background	Eur	Eur	Eur	Eur	Afr. Am	His	Eur	Afr. Am
N	1725	906	754	1529	1041	607	1449	1677
Age	60.98 (8.88)	62.00 (6.53)	62.76 (6.75)	74.61 (7.05)	61.83 (6.67)	60.93 (6.64)	66.32 (6.73)	56.23 (12.31)
Male	843 (48.9)	435 (48.0)	316 (41.9)	1529 (100)	0 (0)	0 (0)	0 (0)	649 (37.17)
BMI	28.11 (4.78)	27.75 (4.25)	27.47 (4.76)	28.00 (4.13)	31.61 (6.43)	29.21 (5.22)	28.84 (5.83)	32.02 (7.37)
*Smoking status*								
Never smoker	720 (41.7)	443 (48.9)	354 (47.0)	478 (31.3)	496 (48.06)	376 (62.46)	766 (53.34)	1490 (85.88)
Ever smoker	1003 (58.2)	463 (51.1)	400 (53.0)	1051 (68.7)	536 (51.94)	226 (37.54)	670 (46.66)	245 (14.12)
Serum creatinine	0.91 (0.27)	0.69 (0.31)	0.85 (0.31)	1.11 (0.45)	0.82 (0.21)	0.73 (0.23)	0.74 (0.14)	0.96 (0.59)
eGFR	86.77 (16.02)	99.77 (21.15)	86.63 (18.94)	69.32 (16.14)	92.31 (19.65)	88.64 (15.39)	83.69 (13.33)	93.57 (22.37)
CKD	99 (5.7)	54 (5.9)	72 (9.5)	407 (26.6)	59 (5.67)	31 (5.11)	82 (5.66)	115 (6.59)
uACR **	6.15 (3.85, 11.97)	9.14 (5.49, 17.98)	8.92 (5.34, 16.53)	NA	NA	NA	NA	5.95 (3.95,13.23)
Microalbuminuria	150 (8.7)	125 (13.8)	104 (13.8)	NA	NA	NA	NA	106 (13.75)
Serum urate	5.37 (1.46)	4.22 (1.49)	4.85 (1.47)	6.13 (1.51)	NA	NA	NA	5.64 (1.70)
Diabetes	158 (9.2)	141 (15.56)	150 (19.89)	235 (15.4)	166 (15.95)	71 (11.70)	96 (6.64)	433 (24.81)
Hypertension	788 (45.7)	516 (55.95)	446 (59.15)	1130 (73.9)	561 (56.21)	207 (35.94)	471 (35.33)	1027 (58.82)
HDL cholesterol	56.47 (14.64)	51.70 (15.79)	53.21 (15.45)	48.81 (12.85)	54.76 (13.78)	51.05 (13.02)	51.38 (11.92)	51.35 (14.73)
Triglycerides **	110 (77, 158)	89.70 (58.30, 140.50)	111.85 (76.50, 116.30)	114 (83, 158)	104 (75, 141)	140 (105, 188)	133 (95, 184)	92 (64, 129)
C-reactive protein **	1.27 (0.63, 2.655)	1.62 (0.83, 3.46)	2.40 (1.06, 4.72)	1.47 (0.75, 3.04)	NA	NA	NA	2.71 (1.18, 5.96)

Population characteristics of all participating studies. The means and standards deviation (SD) are shown for continuous traits, and *N* (%) for categorical traits. **Skewed variables, for which median and (1st, 3rd quartile) are shown. The sample sizes presented here for each of the studies correspond to the number of observations with information on DNAm-predictors, serum-based creatinine kidney traits, chronological age and sex. Age was measured in years at time of participation in study; BMI in kg/m^2^; serum creatinine in mg/dL; eGFR, serum-creatinine-based estimated glomerular filtration rate in mL/min/1.73 m^2^; CKD: prevalent chronic kidney disease, defined as eGFR < 60 ml/min/1.73 m^2^; uACR in mg/g; microalbuminuria was defined as uACR ≥ 30 mg/g; serum urate in mg/dl; prevalent diabetes was defined based on use of glucose lowering drugs or fasting plasma glucose ≥ 126 mg/dl; hypertension defined using the Joint National Committee (JNC) VII definition (blood pressure > 140/90 mm Hg or use of anti-hypertensive medications); HDL cholesterol and triglycerides in mg/dl; C-reactive protein in mg/L; NA denotes the trait was not available. Abbreviations used in ethnic background row: Eur., European ancestry; Afr.Am.., African American; His., Hispanic/Latino. An extended version of this table is shown in Additional file 1: Table S1

DNAm-based age and lifespan predictors were used as independent variables and kidney traits as dependent variables in covariate-adjusted regression models. Study-level results showed associations were slightly attenuated after the inclusion of additional covariates in model 2 (namely BMI, log-transformed triglycerides, HDL, hypertension, smoking status and diabetes) in comparison with model 1 (basic model adjusting for chronological age and sex; Additional file 1: Table S2). Sensitivity analyses showed that “crude” bivariate correlations were largely attenuated after adjustment for chronological age; the introduction of additional variables did not significantly alter the observed effects, despite a slight increase in the coefficient after the introduction of smoking in models with MRS and GrimAge (Additional file 3: Note S1). Estimates from the fully adjusted model were meta-analyzed using inverse-variance weighted fixed-effects and random-effects models. We based our main interpretations on the fixed-effects results; if heterogeneity was large (*I*^2^ > 0.50 and Cochran’s *Q p*_het_ < 0.05), we based our interpretations on the random-effects results (Methods).

### Meta-analysis of associations between DNAm-based predictors and kidney traits

We identified 23 significant DNAm-based predictor-kidney trait associations (*p* < 1.43E−03 and concordant direction of effect across studies; Table 2). Three interesting groups worth further discussion are: (1) PhenoAA, MRS and EEAA were associated with all parameters of poor kidney health (lower eGFR, prevalent CKD, higher uACR or microalbuminuria and higher serum urate); (2) the “first-generation” epigenetic aging markers, where HannumAA was associated with all kidney traits but serum urate, and HorvathAA was only associated with lower eGFR; and (3) an analogous measure to age acceleration in GrimAge was associated with uACR, microalbuminuria and serum urate (Fig. 1). Six associations between DNAm-based predictors and kidney traits were replicated across ethnic groups, and ethnic-specific replication was observed for 16 associations in total.

**Table 2:** Trans-ethnic meta-analyses of associations between kidney traits and DNAm-based age and lifespan predictors

Clock	eGFR					CKD				
	*β*	95% CI	*p*	*I*^2^	*p*_het_	OR	95% CI	*p*	*I*^2^	*p*_het_
HorvathAA	− 0.006	− 0.01, − 0.003	**5.15E−04**	38.696	0.121	1.019	1.003, 1.034	0.016	13.663	0.323
HannumAA	− 0.007	− 0.011, − 0.004	**1.05E−04**	0	0.816	1.033	1.016, 1.05	**8.55E−05**	44.898	0.08
GrimAA	− 0.006	− 0.01, − 0.002	1.94E−03	63.474	0.008	1.027	1.01, 1.044	1.27E−03	77.714	5.2E−05
PhenoAA	− 0.005	− 0.008, − 0.002	**2.62E−04**	0	0.564	1.031	1.018, 1.044	**3.19E−06**	33.885	0.158
EEAA	− 0.008	− 0.012, − 0.005	**2.09E−06**	43.328	0.09	1.038	1.022, 1.055	**3.41E−06**	49.636	0.053
MRS	− 0.117	− 0.158, − 0.075	**4.39E−08**	27.67	0.208	1.784	1.474, 2.159	**2.71E−09**	68.295	0.002^a^
IEAA	− 0.004	− 0.008, 0	0.051	16.08	0.303	1.007	0.99, 1.025	0.427	2.365	0.411

Clock	uACR					Microalbuminuria				
	*β*	95% CI	*p*	*I*^2^	*p*_het_	OR	95% CI	*p*	*I*^2^	*p*_het_
HorvathAA	0.002	− 0.004, 0.008	0.606	0	0.632	1.014	0.992, 1.036	0.223	0	0.703
HannumAA	0.014	0.009, 0.02	**2.04E−06**	0	0.967	1.054	1.032, 1.076	**1.08E−06**	0	0.898
GrimAA	0.029	0.021, 0.037	**1.07E−12**	7.22	0.357	1.106	1.074, 1.138	**7.58E−12**	0	0.59
PhenoAA	0.01	0.005, 0.015	**2.71E−05**	0	0.787	1.035	1.017, 1.053	**8.96E−05**	0	0.612
EEAA	0.013	0.008, 0.017	**4.62E−07**	0	0.989	1.048	1.03, 1.066	**1.42E−07**	0	0.86
MRS	0.252	0.179, 0.324	**1.01E−11**	76.128	0.006^a^	2.238	1.734, 2.889	**6.14E−10**	67.72	0.026^a^
IEAA	0.002	− 0.004, 0.008	0.529	0	0.847	1.015	0.991, 1.04	0.214	0	0.769

Clock	Urate				
	*β*	95% CI	*p*	*I*^2^	*p*_het_
HorvathAA	0.003	− 0.001, 0.007	0.12	0	0.453
HannumAA	0.005	0.001, 0.009	0.011	0	0.919
GrimAA	0.009	0.004, 0.013	**1.17E−04**	56.31	0.057
PhenoAA	0.009	0.006, 0.012	**4.71E−08**	0	0.432
EEAA	0.007	0.004, 0.011	**4.37E−05**	41.60	0.144
MRS	0.115	0.067, 0.162	**2.08E−06**	12.16	0.336
IEAA	− 0.001	− 0.005, 0.003	0.675	0	0.797

Results from trans-ethnic meta-analyses of associations between kidney traits and DNAm-based age and lifespan predictors in up to seven population-based studies. Study-level associations were adjusted for chronological age, sex, BMI, blood lipids, hypertension, smoking and diabetes. Beta coefficients are given as changes in one standard deviation (SD) of the continuous kidney trait. Fully adjusted associations of serum-creatinine-based traits (eGFR, CKD) are based on *N* ≤ 9390 observations, whereas the sample size for urinary albumin-based traits (uACR, microalbuminuria) is *N* ≤ 4406 and for urate *N* ≤ 5769. *I*^2^ is the heterogeneity statistic, and (*Q*) *p*_het_ corresponds to Cochran’s *Q* heterogeneity statistic

Shown in bold are statistically significant associations (*p* < 1.43E-03 and consistent direction of effect across studies) with either no evidence of heterogeneity in the fixed-effects model or supporting findings from the random-effects model

^a^*p*_het_ of MRS with CKD, uACR and microalbuminuria < 0.05, therefore reported association based on significant random-effects models: MRS-uACR: *β* = 0.248 [0.1, 0.397], *p* = 1.061E−03; MRS-CKD: OR = 1.915 [1.316, 2.786], *p* = 6.89E−04; and MRS-microalbuminuria: OR = 2.197 [1.403, 3.439], *p* = 5.82E−04 (Additional file 1: Table S3)

### PhenoAA, EEAA and MRS universally associated with poor kidney health

**Fig. 2:**
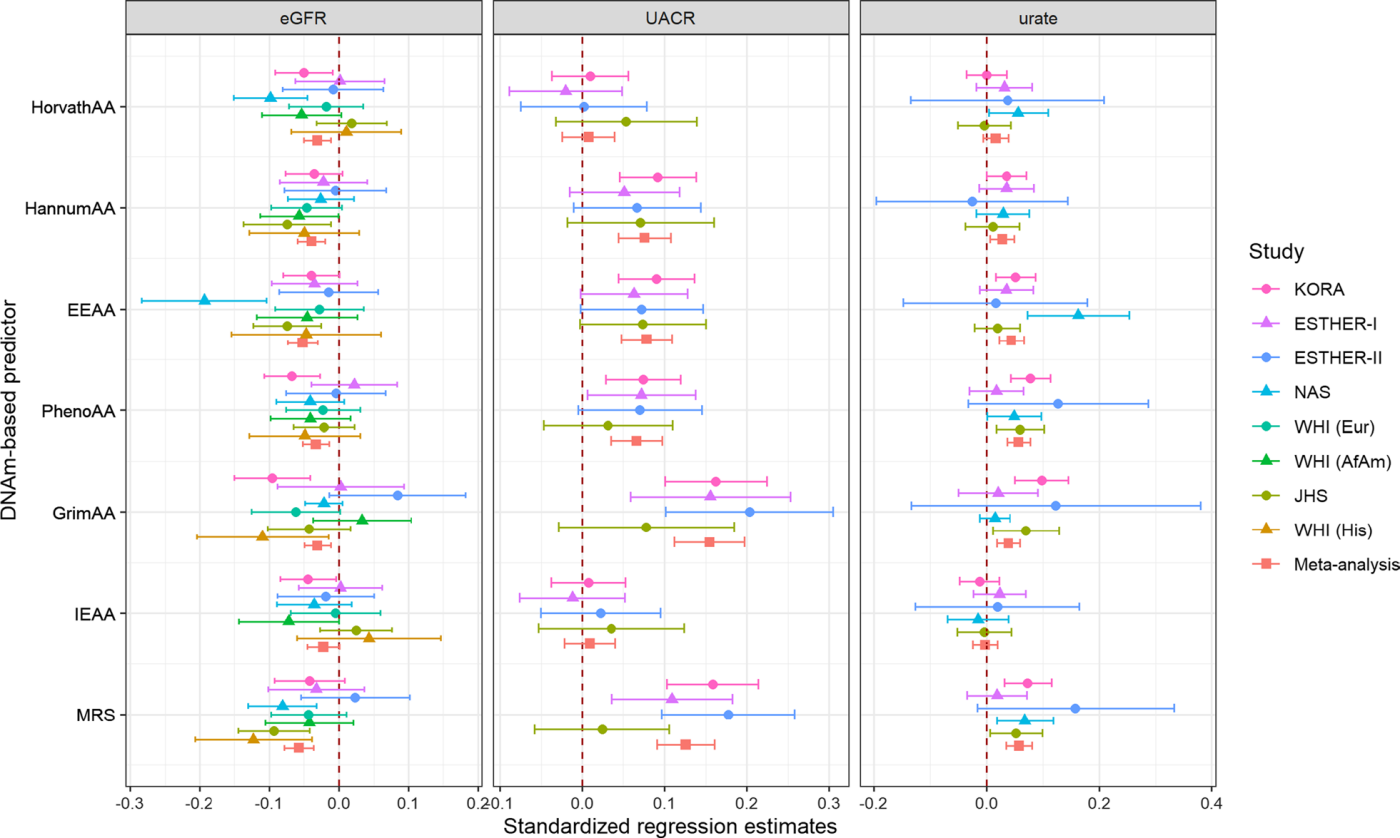
Standardized effect estimates from DNAm-based predictors and kidney traits. Scatter plot showing the effect estimates from the DNAmAge and lifespan predictors across continuous kidney traits for individual studies and trans-ethnic fixed-effects meta-analysis. Effect estimates have been standardized to one SD in both variables to allow for comparison of effect sizes. The legend shows the combination of shape and color coding assigned for the studies

From these three DNAm-based predictors, associations with MRS had the smallest p-values: one MRS unit increase had a − 0.12 (95% CI = [− 0.16, − 0.08]) change in one standard deviation (SD) of log-transformed eGFR (*p* = 4.39E−08) and was associated with 78% [47–116%] increased odds of prevalent CKD (*p* = 2.71E−09) (Table 2). Although high heterogeneity was identified in associations between MRS and CKD, uACR and microalbuminuria, the results from random-effects models (Additional file 1: Table S3) were consistent with the estimates produced by the fixed-effect model: for example, one MRS unit increase was associated with a 0.25 [0.10, 0.40] change in one SD of log-transformed UACR (*p* = 1.06E−03). Figure 2 shows the study-level regression coefficients of the association between the continuous kidney traits and DNAm-based predictors standardized to one SD deviation in both terms to allow for their comparison. While the strength of association with serum urate of these three DNAm-based predictors was similar, MRS and EEAA had the largest effects on eGFR (Fig. 2, standardized effects in Additional file 1: Table S4). Similar observations were done for the binary kidney traits (Additional file 2: Fig. S1).

**Fig. 3:**
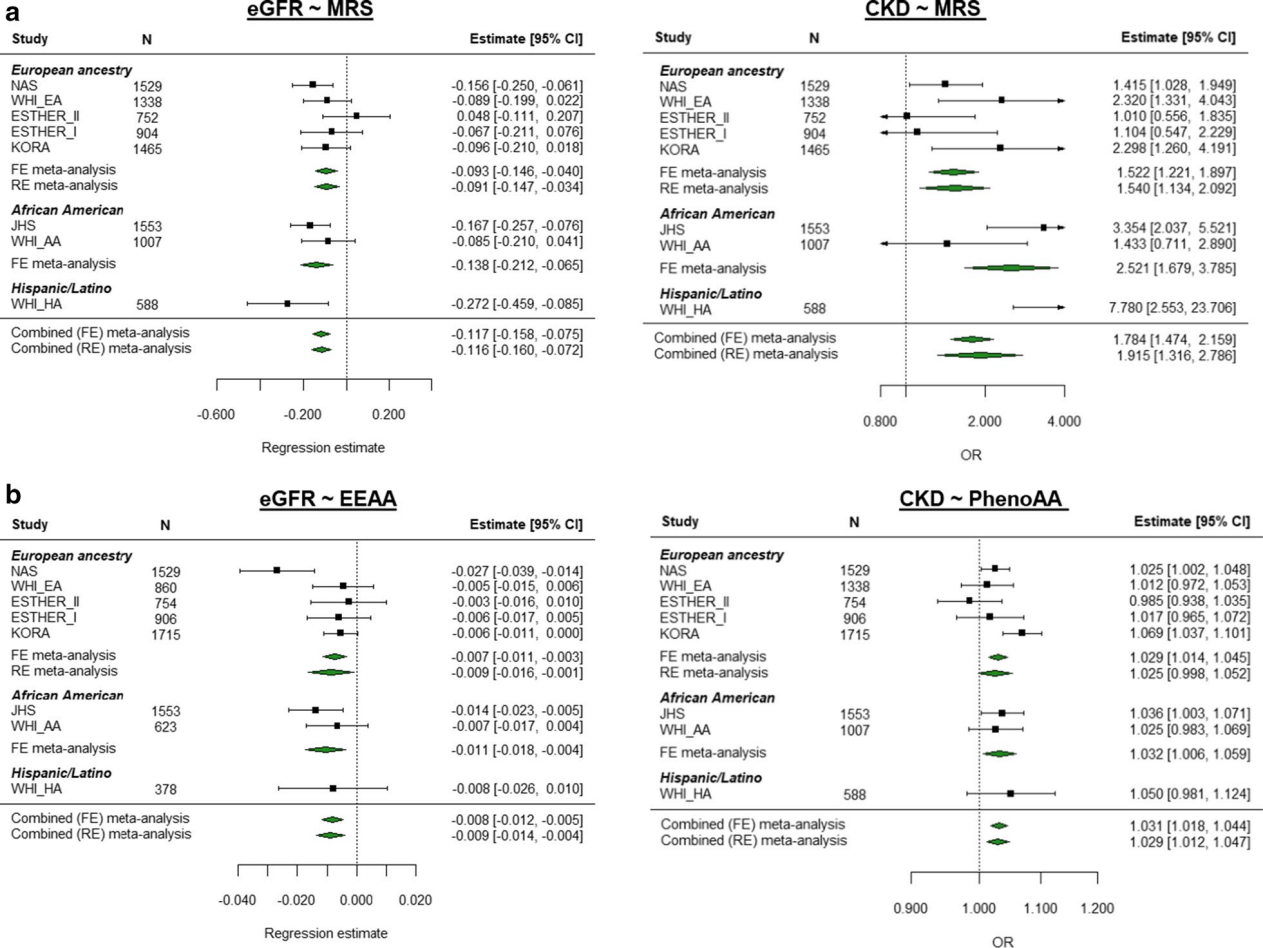

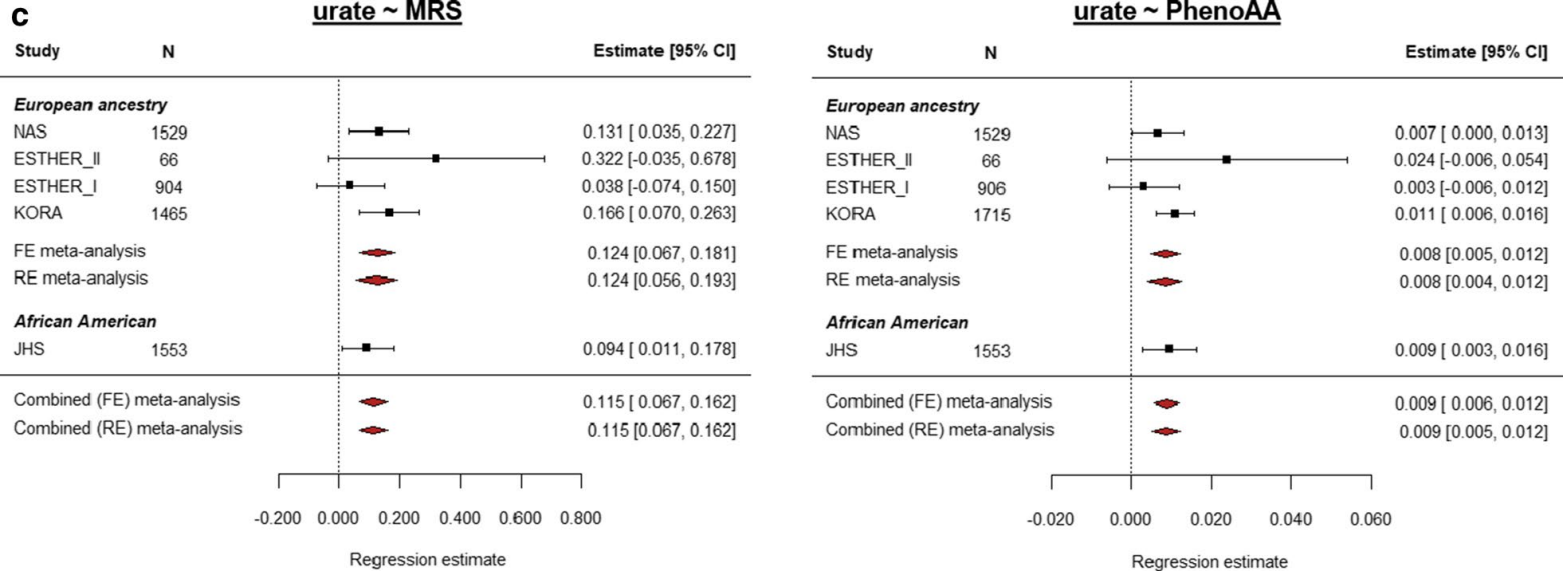
Multivariate regression meta-analysis of association between kidney traits and MRS, EEAA and PhenoAA. Multivariate regression models were used to assess the relationship between kidney traits and age acceleration as measured by seven DNAm-based predictors of age and/or lifespan, including Zhang’s 10-CpG mortality risk score (MRS), PhenoAge and extrinsic epigenetic age acceleration (EEAA). Results from the fully adjusted model included chronological age, sex, BMI, log-transformed triglycerides, HDL, hypertension, smoking status and diabetes as covariates, and baseline eGFR for serum urate analyses. Inverse-variance weighted fixed-effects models meta-analysis was conducted, where if heterogeneity was observed ([*Q*] *p*_het_ < 0.05), a random-effects model was further interpreted. Individual panels show forest plots with the study-level and meta-analytic results for each association between kidney trait and DNAm-based predictor from the fully adjusted model. The rows correspond to the different studies and the sample size for each analysis (*N*). For eGFR and urate, the regression estimates represent the change in one standard deviation of the kidney trait per unit change in the MRS or per one year of age acceleration for PhenoAA and EEA. For CKD, the estimate column corresponds to the odds ratio (OR). The *x*-axis shows the estimates obtained from either the regression model (for single studies, data point shape is a black square) or the meta-analytic estimate (data point shape is a green diamond for serum creatinine-based traits and a red diamond for urate) with their 95% CI. Estimate: regression coefficient for the continuous traits and OR for CKD, 95% CI: 95% confidence interval of the estimate

Figure 3 shows results from ethnic-specific meta-analyses significant in both European ancestry and African American meta-analyses (*p* < 1.43E−03, Additional file 1: Table S3). MRS stands out for its replication across all subgroups, including the small Hispanic/Latino cohort (Fig. 3a, study-level results in Additional file 1: Table S2). The eGFR-EEAA and CKD-PhenoAA effects were also replicated at the Bonferroni-corrected significance level across ethnic-specific meta-analyses (Fig. 3b), whereas their “complementary” associations (CKD-EEAA and eGFR-PhenoAA) were nominally significant (Additional file 2: Fig. S2A). Further effects observed in both European ancestry meta-analysis and the African American cohort were the associations of higher serum urate with MRS and PhenoAA (Fig. 3c). On the other hand, the associations between EEAA, PhenoAA and MRS with higher uACR (and prevalent microalbuminuria) identified in the trans-ethnic meta-analysis were mostly driven by the effects from the European ancestry cohorts (Additional file 2: Figs. S3 and S4), as well as that of serum urate and EEAA (Additional file 2: Fig. S5A). Most notably, the association between uACR and MRS was replicated at the Bonferroni-corrected level in two of the European ancestry cohorts (KORA and ESTHER-II), and nominally significant in the third one (Additional file 1: Table S2).

### Associations with “first-generation” DNAm clocks and GrimAge

HorvathAA, age acceleration measured by the “first-generation” DNAm-based predictor HorvathAge, was exclusively associated with low eGFR: a one year difference between DNAm-estimated age and chronological age was associated with a − 0.006 [− 0.01, − 0.003] change in log-transformed eGFR (*p* = 5.15E−04, Table 2). HannumAA, another “first-generation” DNAm-based age predictor, was also associated with eGFR; the strength of the association with both “first-generation” DNAm-based predictors was similar (Fig. 2). HannumAA was additionally associated with CKD, uACR and microalbuminuria (Additional file 2: Figs. S2–S4B). While the effects observed for HannumAA with eGFR and CKD were robustly replicated by cohorts with African American participants (Additional file 2: Fig. S2B), the association between HorvathAA and eGFR was mostly driven by studies with European ancestry (Additional file 2: Fig. S2C).

A year of GrimAge acceleration was associated with an increase of 0.03 SD of log-transformed uACR and a 10.6% increase in the odds of having microalbuminuria, both early markers of renal damage (Additional file 2: Figs S3–S4C). Likewise, a one-year difference in GrimAge was associated with higher serum urate levels, a risk factor for cardiorenal disease (Additional file 2: Fig. S5B). The GrimAA effects on uACR were the largest across DNAm-based predictors and kidney traits (Fig. 2), and a similar effect was observed for microalbuminuria (Additional file 2: Fig. S1).

### Associations with categorical MRS and GrimAge components

In the categorical MRS, where risk groups were defined based on the number of CpGs with methylation levels beyond pre-defined high-risk thresholds, a 0.431 (95% CI = [0.29, 0.57]) SD increase in log-uACR was observed for individuals with > 5 “aberrantly” methylated CpGs (high risk MRS) compared to those with 0–1 aberrantly methylated loci (low risk MRS) (*p* = 4.01E-09, Additional file 2: Fig. S6A). Similar associations were observed for microalbuminuria (OR = 2.075 [1.582, 2.722], *p* = 1.36E-07; Additional file 1: Table S3). The moderate MRS category (defined as those with 2–5 “aberrantly” methylated loci) was also associated with serum urate (Additional file 2: Fig. S6B). These effects were mostly driven by European ancestry studies, where effects had a similar strength of association and replicated within this group (Additional file 2: Fig. S7). None of these effects replicated in the African American cohort, perhaps due to the smaller sample size and reduced statistical power.

In regard to the secondary analyses with the eight DNAm-based components that constitute GrimAge, we found associations for three of them. DNAm-estimated adrenomedullin (DNAmADM), plasminogen-activation inhibition 1 (DNAmPAI1) and pack years (DNAmPACKYRS) were positively associated with higher uACR, higher serum urate levels and microalbuminuria (Additional file 1: Table S5). Of note, the effects identified for serum urate replicated in both the European-ancestry analyses and in the African American cohort (Additional file 1: Table S2), whereas those in uACR and microalbuminuria were mostly replicated within the European studies.

## Discussion

We identified 23 associations between kidney traits and DNAm-based predictors of aging and/or mortality in a large trans-ethnic meta-analysis from up to seven multi-ethnic population-based cohorts. PhenoAA, MRS and EEAA were associated with all parameters of poor kidney health (lower eGFR, prevalent CKD, higher uACR or microalbuminuria and higher serum urate). Distinct patterns of association were observed with age acceleration in the “first-generation” clocks (Hannum and Horvath) and an analogous measure in GrimAge: HorvathAA was only associated with lower eGFR, while HannumAA was associated with all kidney traits but serum urate. Finally, GrimAA was associated with uACR, microalbuminuria and serum urate.

Sensitivity analyses showed that the inclusion of chronological age in a “crude” model lead to large attenuation of the correlation between DNAm-based predictors and kidney traits, as expected when adjusting for variables confounding the exposure-outcome association. The posterior introduction of additional variables did not further alter the observed effects. A slight increase in the coefficient after the introduction of smoking was observed for MRS and GrimAge, which may be explained in general by the strong correlation between smoking and DNAm/DNAmAge acceleration in blood [31–34], and specifically in relation to the these two markers as they either directly incorporate cigarette smoking into its formulation [19] or capture the effects of cigarette smoking [20].

From the three “universally” associated DNAm-based predictors, MRS was robustly associated with multiple kidney phenotypes, some of which replicated across ethnic-specific analyses (i.e., eGFR, prevalent CKD and urate). Together with EEAA and GrimAA, MRS showed the largest effects in comparison with the other DNAm-based predictors. Moreover, MRS moderate and high risk categories [20] were also associated with eGFR and markers of kidney injury in the secondary analyses. Recent evidence suggests the MRS is a DNAm-based biomarker which reflects mortality risks by capturing the effects of oxidative stress and systemic inflammation, as well as inflammation-driven changes in immune cell counts—all mechanisms shared by numerous chronic diseases [35–38]. Particularly in kidney aging, mitochondrial dysfunction, uremia-induced epigenetic changes and the production of reactive oxygen species in the glomeruli that lead to barrier function impairment and albuminuria are all mechanisms promoting oxidative stress [8, 39–41]. These factors may thus explain the associations between kidney traits and MRS here identified. The grounding of the MRS in inflammation and oxidative stress mechanisms may also explain the predictive power of this predictor in regard to outcomes from cancer to cardiovascular disease mortality [20, 21].

EEAA and PhenoAA, also associated with all kidney traits, are both extrinsic aging measures (i.e., tracking changes in blood cell composition) that also capture (intrinsic) aging-related physiological dysregulation [12, 18]. EEAA, identified as a measure of immune system aging, is considered better at predicting age-related decline of tissue performance [12, 14, 18]. Increased allostatic load, activation of stress and pro-aging pathways, impairment of protective pathways as well as exogenous lifestyle and environmental factors are all factors driving premature aging [17, 39]. Immunosenescence [42, 43], systemic low-grade inflammation ('inflammaging') [44] and oxidative stress [37, 39] contribute to an increased allostatic load and are mechanisms present in kidney aging [17, 45]. In particular, the immune system plays an important role in the incidence, development and resolution of renal disease [43]. A signature of 447 genes involved in renal aging further confirmed the relevance of cellular pathways common to immune function and renal physiological decline [46]. Likewise, CKD patients show signs of premature immunological aging (such as poor naïve T-cell frequency and reduced thymic output), a status induced by ureamia (high concentrations of serum urate in blood) that is associated with poor clinical outcomes [47]. At the cellular level, telomere attrition [39, 48] and the cytokine secretory profile of senescent cells promote inflammation and lead to fibrotic damage [37, 39, 43, 45], further linking inflammaging to renal dysfunction [9]. Our findings, in line with the positive association between PhenoAge and albumin excretion rate identified in 499 subjects with type 1 diabetes [49], may be thus explained by the relationship between these DNAm-based predictors and the aging-related changes to the immune system, low-grade chronic inflammation and oxidative stress that impact renal disease [37, 47]. Moreover, EEAA, PhenoAge and the MRS have shown a stronger predictive association with time to death than HannumAA, HorvathAA and IEAA, suggesting they better reflect mortality risks associated with biological aging [16, 20]—and based on our findings, also better reflect immune system changes associated with kidney aging. The correlation between these DNAm-based predictors and poor kidney health may also explain their strong associations with mortality, as the ensuing contribution of renal disease to physiological dysregulation may increase mortality risk [6, 7, 39, 50].

Distinct patterns of association were identified with two of the “first-generation” clocks, HorvathAA and HannumAA. HorvathAA, thought to track cell-intrinsic aging (e.g., epigenetic stability mechanisms, cell growth and survival as well as organismal development) [11, 12, 16, 18], was exclusively associated with eGFR in our study. The statistical power derived from the larger sample size in our study likely explains this positive association, unlike prior studies reporting null findings with eGFR [49, 51]. Considering tissue from individuals with renal disease was included in the development of this pan-tissue marker [11], this epigenetic marker reflects alterations in ubiquitous cell-intrinsic pathways that our findings suggest may be relevant for renal (glomerular) function. Genome-wide association studies of the Hannum and Horvath DNAm-based predictors have shown that, even though they capture different aspects of aging, both markers are influenced by genes associated with metabolic and immune system pathways [52, 53]. Consequently, these DNAm-based predictors may also somewhat be reflective of the immune molecular mechanisms previously described. An additional factor of potential relevance for the eGFR-HorvathAA association may be aberrant glucocorticoid signaling, given its potentially pathogenic role in renal function [54] and the enrichment of glucocorticoid response elements in this DNAm marker [29].

HannumAA, associated with lower renal function and markers of early renal damage (uACR and microalbuminuria) in our study, is also strongly correlated with blood cell counts [16, 23] and is sensitive to environmental influences [15, 53]. Moreover, it has also been associated with higher levels of inflammatory biomarkers, creatinine and certain lipid classes in individuals of European ancestry [14, 15, 55]. Like other extrinsic measures, Hannum seems to be a better marker for later-life diseases and mortality than Horvath [22] and has even been proposed as a prognostic marker of pathological metabolic processes [56]. HannumAA in cancerous kidney tissue vs normal samples has also been reported [10], thus offering further evidence on the relevance of our findings in blood to renal disease.

The association between one-year difference in the GrimAge predictor and higher uACR, serum urate and microalbuminuria—but not eGFR or prevalent CKD—suggests this DNAm-based predictor might be more sensitive to systemic inflammation and early renal damage. Albuminuria changes can occur before eGFR decline in early kidney disease [6]: early structural glomerular lesions in patients with normal eGFR are better correlated with changes in uACR than with GFR decline, where the latter might not be present yet [57]. Moreover, albuminuria is a predictor of CKD progression and mortality independently from eGFR changes, which suggests they represent two independent mechanisms underlying renal disease progression [6, 7]. Our findings are in line with prior reports of GrimAge association with albumin excretion in T1D patients and non-diabetic subjects [19, 49], where lower power may explain the null findings reported in [51]. Asymptomatic hyperuricaemia, or high serum urate levels, is involved in pro-inflammatory mechanisms and is associated with a high risk of cardiovascular and renal disease [8]. Serum urate, both in its crystal and soluble forms, activates innate immunity and triggers DNAm epigenetic mechanisms (e.g., promoting cytokine secretion, pro-inflammatory pathways including oxidative stress) leading to persisting inflammation and an increased allostatic load [8, 39]. These effects may, in turn, explain our findings in relation to GrimAge. A high degree of heterogeneity in the eGFR- and CKD-GrimAA analyses was observed, similarly to reports in prior studies [58, 59].

Overall, the observed associations did not show a clear pattern across kidney traits, thus supporting the proposed notion that the existing DNAm-based predictors might reflect different aspects of biological aging. This is in line with their differential association with risk factors, intermediate phenotypes and diseases [12, 58, 60, 61], and their inclusion of non-overlapping CpGs sets [19, 20, 60, 62]. The CpG overlap between DNAm-based predictors was assessed in detail by Liu et.al, where the lack of CpG overlap may be explained by the redundancy of the methylome: CpGs selected in the construction of different DNAm predictors may represent different aging hallmarks or pathways, despite the potential biological similarities of their genomic regions [62]. Our findings are also consistent with the many associations observed with EEAA and other blood immune system correlated DNAm-based predictors [12], and with the lack of associations with IEAA observed in prior studies [52, 61].

DNAm-estimated adrenomedullin (DNAmADM), plasminogen activator inhibitor-1 (DNAmPA1) and smoking pack years (DNAmPackYears) were positively associated with decreased renal function. Consistent with our findings, patients with chronic cardiorenal diseases have higher blood levels of ADM [63, 64] and PAI-1 [65–67], where the first is a potential biomarker of CKD progression [64, 68] and the latter a risk factor for cardiorenal disease [66, 69]. Several factors involved in kidney disease pathogenesis (e.g., oxidative stress, inflammation) induce PAI-1 expression (66, 70, 71), which in turn has been linked to fibrosis, glomeruli damage and other pathogenic mechanisms in renal disease [65, 67], as well as to thrombosis and an increased hypercoagulable state—a shared phenotype of inflammaging [37] and renal disease [72]. Moreover, the effects of smoking in DNAm [31, 32] and their association to DNAmAge acceleration in blood are well known [33, 34]. PhenoAge and MRS capture effects of cigarette smoking [18, 20, 33, 58, 60], whereas GrimAge specifically incorporates cigarette smoking into its formulation [19]. Cigarette smoking has been associated with renal function decline and increased inflammation [73], where several mechanisms explaining the negative effects of smoking on renal function (e.g., oxidative stress, endothelial dysfunction, immune function modulation) contribute to renal disease progression [74]. Overall, our findings are in line with the roles described in the literature for the studied DNAm-estimated markers, and further support the notion that associations between epigenetic aging and health outcomes may be mediated by age-related pro-inflammatory mechanisms [75]. Moreover, they suggest DNAm-based estimates might prove to be valuable proxies in settings where such variables are not available (e.g., limitations in the clinical use of ADM [68] and self-reported smoking [34]).

Strengths of this work are the large sample size and inclusion of multiple independent studies involving multi-ethnic populations. We comprehensively addressed biological aging and lifespan as predicted by DNAm and assessed multiple kidney traits reflecting different aspects of renal health. Our results in regard to PhenoAge and GrimAge represent confirmatory findings to some extent, given that renal function variables were included in the derivation of these algorithms [18, 19]. Of note, although the MRS was derived using data of two of the cohorts included in this study, it has been independently validated [20, 21] and the replication of its associations across multiple cohorts suggest our findings are not a product of data overfitting. All in all, our study meets the considerations proposed by a recent literature review and meta-analysis on the topic [22].

Limitations of this study include the estimation of DNAm markers in blood samples rather than renal tissue, although there is currently no epigenetic age predictor derived in kidney tissue. Age-related methylation changes can be tissue-specific [76] and show inter-individual variation [46], yet associations between eGFR and DNAm in blood have been demonstrated to be relevant to kidney traits [46, 77]. Moreover, this remains the only viable approach for research conducted in population-based studies, where taking renal biopsies from participants is not done due to practical and ethical considerations. Despite the bias inherent to the calculation of eGFR using equations that systematically produce higher values for individuals identified as black [2, 78, 79], associations with trans-ethnic replication in our study featured lower eGFR (CKD). Nevertheless, future kidney research would benefit from the development and use of methods relying on filtration markers independent from muscle mass and/or moving beyond race as a variable [78]. The lack of trans-ethnic replication of all associations may be explained by multiple factors, most notably the smaller sample sizes from non-European studies, or ethnic biases in DNAm-based predictors (as those reported for PhenoAge in [80]). Future studies using larger, homogeneous sample sizes from diverse ethnic groups are needed to address the generalizability of our findings, and to interrogate the contributions of environmental and social determinants of health disparities in epigenetic aging. Our models assumed a linear relationship in the age range studied here, and residual confounding after adjustment for the covariates included in our regression analyses is a possibility. Another potential limitation is that the cross-sectional nature of the study does not allow to draw conclusions on temporal relationships between DNAm and renal phenotypes, although DNAm patterns reflect lifetime environmental exposures and genetic factors. Our findings do not provide a mechanistic or causal explanation for renal aging and blood epigenetic aging markers, but should be considered hypothesis-generating research. Preliminary results from the largest genome-wide association (GWAS) study of DNAm-based aging and lifespan predictors found no evidence of causal effects on renal outcomes (uACR, eGFR, albuminuria and serum urate, among 150 studied traits) [81]. Nevertheless, DNAm-based aging/lifespan signatures could still be a valuable biomarker of kidney disease prognosis, risk stratification or kidney-related outcomes. Future research should aim to expand our understanding of epigenetic aging in chronic diseases and on the clinical utility of the DNAm-based predictors.

## Conclusion

In this study of multi-ethnic population-based cohorts, kidney traits were robustly associated with DNAm-based aging and lifespan predictors measured in whole blood, as well as with some secondary DNAm-estimated markers. Our findings are consistent with a body of literature on the role immunosenescence, inflammaging and oxidative stress play in renal function and damage, as well as offer evidence on the relevance of cell-intrinsic aging mechanisms. DNAm age and lifespan predictors seem to capture the contribution of multiple CpGs to pathological changes common to systemic inflammation and renal disease, highlighting the systemic nature of age-related physiological functional decline. Future research in longitudinal studies is required to evaluate the translational value of our findings as either prognostic biomarkers for disease progression and mortality, or as means to enhance risk stratification; functional studies to explore the complex physiological interplay between epigenetic mechanisms and biological aging are also warranted.

## Methods

### Study design

The association between kidney traits as dependent variables and DNAm aging/lifespan predictors as independent variables was modeled using linear regression following a meta-analytic approach (Fig. 1). Study-level results from up to seven studies were included in the meta-analyses: four studies with participants of European ancestry, one study of African American participants and three substudies from the WHI with European American, African American and Hispanic/Latino participants. The studies were KORA (Kooperative Gesundheitsforschung in der Region Augsburg), NAS (Normative Aging Study), ESTHER (Epidemiologische Studie zu Chancen der Verhütung, Früherkennung und optimierten THerapie chronischer ERkrankungen in der älteren Bevölkerung), WHI (Women's Health Initiative) and the Jackson Heart Study (JSH). The ESTHER, NAS and WHI studies contributed multiple sets of data that were analyzed separately: data sets from ESTHER corresponded to two surveys with non-overlapping sets of participants, whereas data from NAS were longitudinal and collected over consecutive examinations and analyzed taking into account these repeated measures. Further details on the data collection and methods used in each study are available in Additional file 3.

### Outcome definition

Serum creatinine values obtained with a Jaffé assay before 2009 were calibrated by multiplying by 0.95 [82] and used to calculate estimated glomerular filtration rate (eGFR) as per the CKD-EPI equation [2] in its implementation in the R package *nephro* [83]. Prevalent chronic kidney disease (CKD) was defined as eGFR < 60 ml/min/1.73 m^2^ [84]. eGFR and urinary albumin-creatinine ratio (UACR) were log transformed prior to statistical analysis. Microalbuminuria was defined as uACR ≥ 30 mg/g. Serum urate was also studied.

### DNAmAge assessment

Methylation was measured using the Illumina Infinium HumanMethylation450K or EPIC array in whole blood and used to estimate measures of DNAmAge and mortality (additional details on each predictor are described in Additional file 3: Note S2). Five DNAmAge and lifespan predictors were calculated using the online DNAm Age calculator (https://dnamage.genetics.ucla.edu/) [11]: Hannum’s estimate (HannumAge), ExtrinsicAge (EEAA) [10], Horvath’s estimate (HorvathAge) [11], PhenoAge [18] and GrimAge [19]. IEAA, a marker capturing cell-intrinsic aging properties that are independent of blood cell types, was derived by regressing HorvathAge on cell counts [10]. Quality control was conducted as in previous meta-analyses of epigenetic measures [16], with exclusion of individuals with mismatching predicted and reported sex data. Age acceleration (AA) measures were calculated in each study as the difference between the predicted DNAmAge and chronological age, with chronological age included in all models as an adjustment for known chronological age effects across the lifespan. Defining age acceleration as the difference, rather than the residual of chronological age regressed on epigenetic age, has advantages as it is an individual measure as opposed to a population measure, and is not defined to have mean 0 in each population as is the case for the residual measure. A sixth measure, the 10-CpG-based epigenetic mortality risk score (MRS) in its continuous form was calculated as the sum of the methylation β values multiplied by the regression coefficients of each of the ten CpGs for all-cause mortality, as described in [20].

Further measures of epigenetic aging were included in the secondary analysis: the risk-level MRS variable was built based on the cumulative number of “aberrantly” methylated CpG sites, defined by the cut-offs derived from the 4th quartile of the CpG positively correlated with mortality (cg08362785) and the 1st quartile of the other nine loci defined in [20]. Participants were then assigned to one of three risk levels based on the total number of “aberrantly” methylated CpGs: low risk, MRS = 0–1; moderate risk, MRS = 2–5; and high risk, MRS > 5. Finally, to better understand GrimAge, we also included in the analysis its eight underlying traits (smoking pack-years, adrenomedullin, beta-2 microglobulin, cystatin C, growth differentiation factor 15, leptin, plasminogen activation inhibitor 1, tissue inhibitor metalloproteinase 1) [19] if a renal phenotype was associated with GrimAge.

### Statistical analysis

Linear and logistic regression models were run with kidney traits as outcomes and measures of DNAm-based age acceleration and lifespan as predictors, including covariates to adjust for potential confounding by biological and technical factors. Chronological age and sex were included in a basic model, whereas additional adjustment for BMI (kg/m^2^), log transformed triglycerides, HDL, hypertension, smoking status (current/ever, never) and diabetes was done in the fully adjusted model. Linear regression models for serum urate additionally adjusted for baseline eGFR. Details on the study-specific definition or exclusion of variables, as well as additional information on all of the cohorts, are given in Additional file 3: Note S3. Leukocyte count (either measured or estimated by the Houseman approach [85]) was additionally included in the regression models for Horvath’s estimate as to obtain the intrinsic age acceleration measure (IEAA). In the secondary analyses, the aforementioned covariates from the basic and the fully adjusted models were used, with the exception of no smoking adjustment for DNAm-predicted pack years. All measures of association between epigenetic markers and continuous renal traits (eGFR, uACR, urate) were standardized to the standard deviation of the given renal trait as to obtain estimates comparable across renal traits.

All outcomes were available in at least one cohort of European ancestry and African American studies (Additional file 1: Table S1), although eGFR and CKD were the only outcomes reported by all participating studies. Each cohort provided regression estimates and standard errors, which were pooled using inverse-variance fixed-effects and random-effects models. Between-study heterogeneity was assessed using Cochran’s *Q* and *I*^2^ statistics. High heterogeneity was defined as *I*^2^ > 0.50 and (*Q*) *p*_het_ < 0.05. If high heterogeneity was detected in the fixed-effects model, the random-effects model was interpreted. All statistical analyses were conducted using R version 3.5.3 [86], where meta-analyses were conducted using the *metafor* package v2.0 [87]. Multiple testing was addressed by correcting the significance level for the total number of statistical tests (i.e., Bonferroni correction, 0.05/7 epigenetic markers * 5 renal traits). Associations were considered significant if *p* < 1.43 E−03 in the trans-ethnic meta-analysis and if they had consistent direction of effect across studies. Ethnic-specific replication was defined as associations with consistent direction of effect reaching nominal statistical significance (*p* < 0.05) in two or more studies.

## Supplementary Information

**Additional file 1: Tables S1–S5** showing the results from all conducted analyses across renal parameters and DNAm-based predictors, providing both estimates from individual studies and meta-analyses.

**Additional file 2: Figures S1–S7** showing forest plots for all significant associations not presented in the main figures, as well as secondary traits (categorical MRS), comparison of MRS and DNAmAA effect sizes across renal traits.

**Additional file 3: Notes** including additional information on sensitivity analyses, the derivation of the DNAm-based predictors, cohort-specific details (background and study design, as well as data collection and pre-processing), and all legends to supplemental tables and figures.

## Data Availability

The dataset(s) supporting the conclusions of this article is(are) included within the article (and its additional file(s)). The informed consent given by the study participants does not cover posting of participant level phenotype data in public databases. Pre-existing data access policies for each of the five studies state research data requests can be submitted to each steering committee. Study-specific details regarding such requests are described in the next points: KORA data are available upon request from KORA Project Application Self-Service Tool (https://epi.helmholtz-muenchen.de/); data requests can be submitted online and are subject to approval by the KORA Board. ESTHER data are not allowed to be publicly available due to restrictions of informed consent. However, the use of the data for collaboration projects has been and will remain the approach for data sharing. Data used in this analysis were produced and used in accordance with the policies of the Jackson Heart Study under contracts from the National, Heart, Lung and Blood Institute and are not the domain of the authors but that of the Jackson Heart Study. These data are available to other researchers for purposes of reproducing the results or replicating the procedures by submitting a manuscript proposal to the Jackson Heart Study at jhspub@umc.edu, as described at https://www.jacksonheartstudy.org/Research/Publications#submitmanuscript. Data updates for the Jackson Heart Study are also deposited regularly in the National Institutes of Health data repositories, dbGaP (https://www.ncbi.nlm.nih.gov/gap/) and BioLincc (https://biolincc.nhlbi.nih.gov/home/). NAS data are available on request due to privacy/ethical restrictions. Data that support the findings of this study are available from AAB upon reasonable request. The DNA methylation datasets for WHI are publicly available under dbGAP access number phs000200.v10.p3 or upon request to www.whi.org. WHI datasets are also available through BioLINCC https://biolincc.nhlbi.nih.gov/studies/whi_ctos/. Scripts used in data processing and statistical analyses have been made publicly accessible at https://ascgitlab.helmholtz-muenchen.de/pamela.matias/dnam-aging-in-kora-f4 and https://ascgitlab.helmholtz-muenchen.de/pamela.matias/kidney-dnamage.

## Abbreviations

AA: Age acceleration defined as the difference between chronological and DNAmAge
HorvathAA: Age acceleration calculated with Horvath’s DNAmAge predictor
HannumAA: Age acceleration calculated with Hannum’s DNAmAge predictor
CKD: Chronic kidney disease
CpG: Cytosine-phosphate-guanine dinucleotide
DNAm: DNA methylation
DNAmAge: Estimated “biological” age using DNA methylation information, also known in the literature as epigenetic age
EEAA: “Extrinsic epigenetic age acceleration,” calculated with Hannum’s DNAmAge predictor
eGFR: Estimated glomerular filtration rate
ESTHER: Epidemiologische Studie zu Chancen der Verhütung, Früherkennung und optimierten THerapie chronischer ERkrankungen in der älteren Bevölkerung
GrimAA: Measure analogous to other age acceleration markers calculated with DNAm GrimAge predictor
JHS: Jackson Heart Study
KORA: Kooperative Gesundheitsforschung in der Region Augsburg
IEAA: Intrinsic epigenetic age acceleration
MRS: Epigenetic mortality risk score
NAS: US Department of Veterans Affairs’ Normative Ageing Study
PhenoAA: Age acceleration calculated with DNAm PhenoAge predictor
uACR: Urinary albumin-to-creatinine ratio
WHI: Women’s Health Initiative
