# Supplementary material for: DNAm-based signatures of accelerated aging and mortality in blood are associated with low renal function
Source: Clin Epigenetics. 2021 Jun 2;13:121. doi: 10.1186/s13148-021-01082-w (PMC8170969; doi:10.1186/s13148-021-01082-w)

DNAmAge acceleration and renal function

Suppl. Figures

Suppl. Figure 1. Odds Ratios from DNAm predictors and binary kidney traits

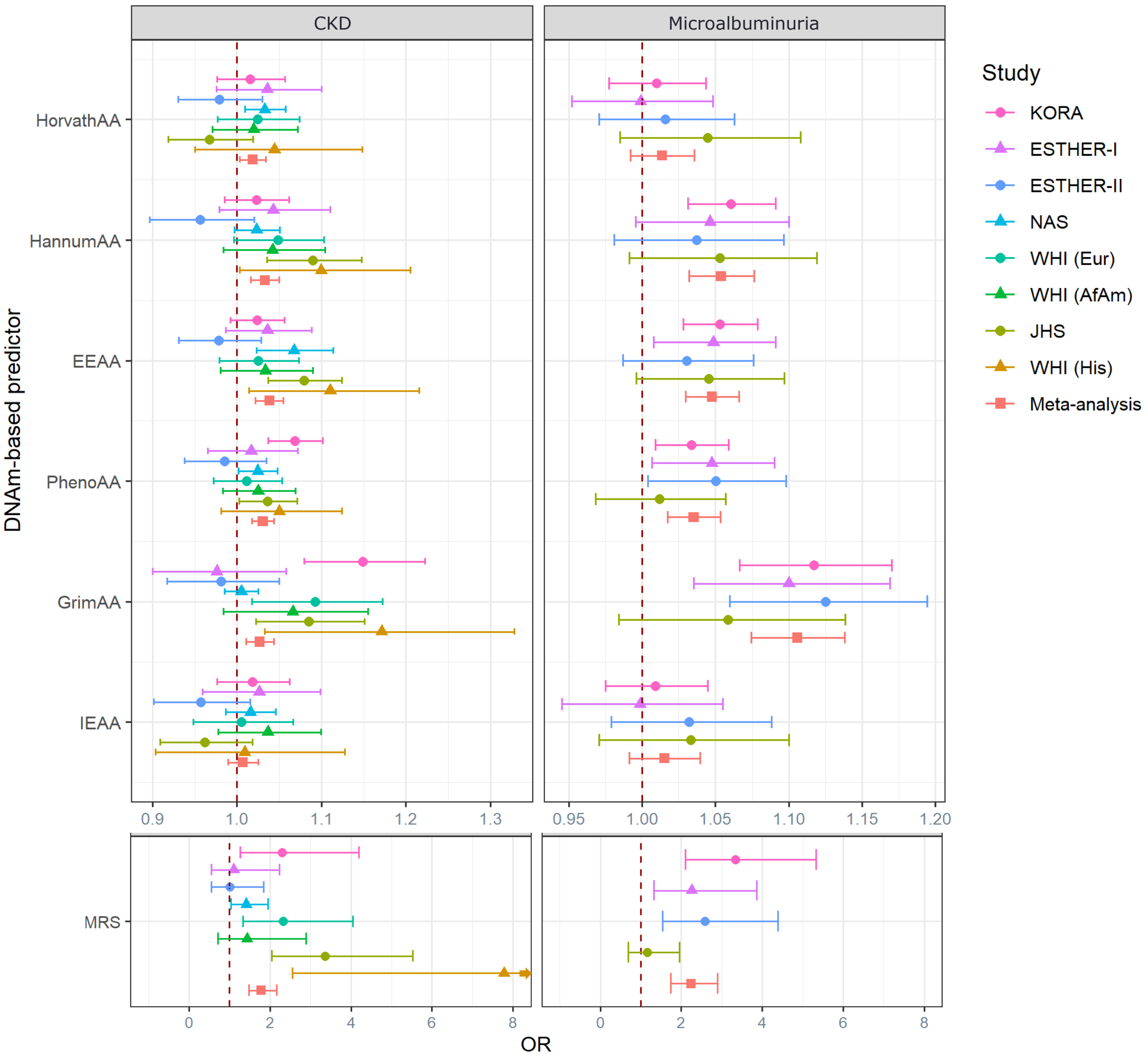

Suppl. Figure 2. Forest plots of associations with eGFR and CKD

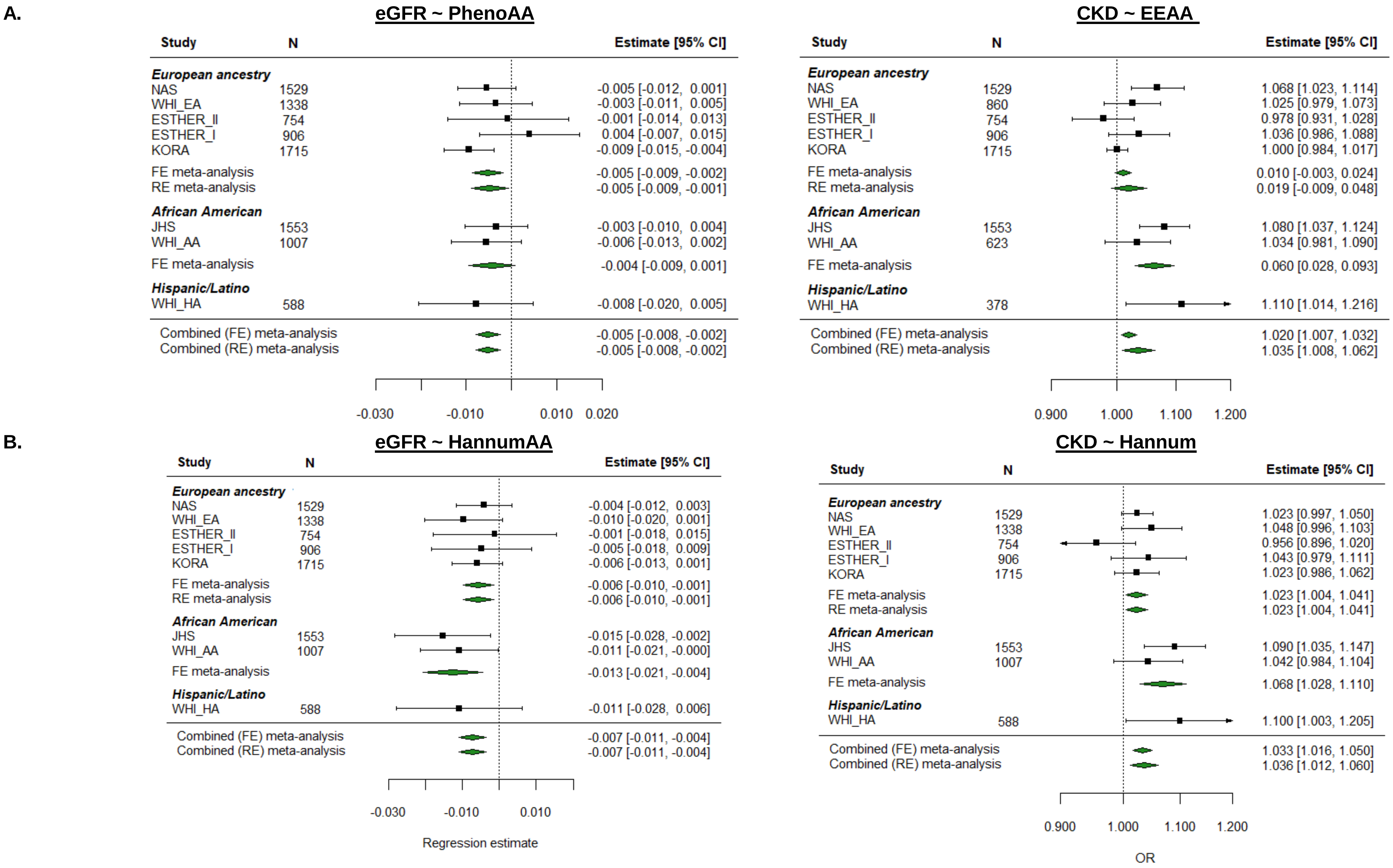

C. **eGFR ~ Horvath**

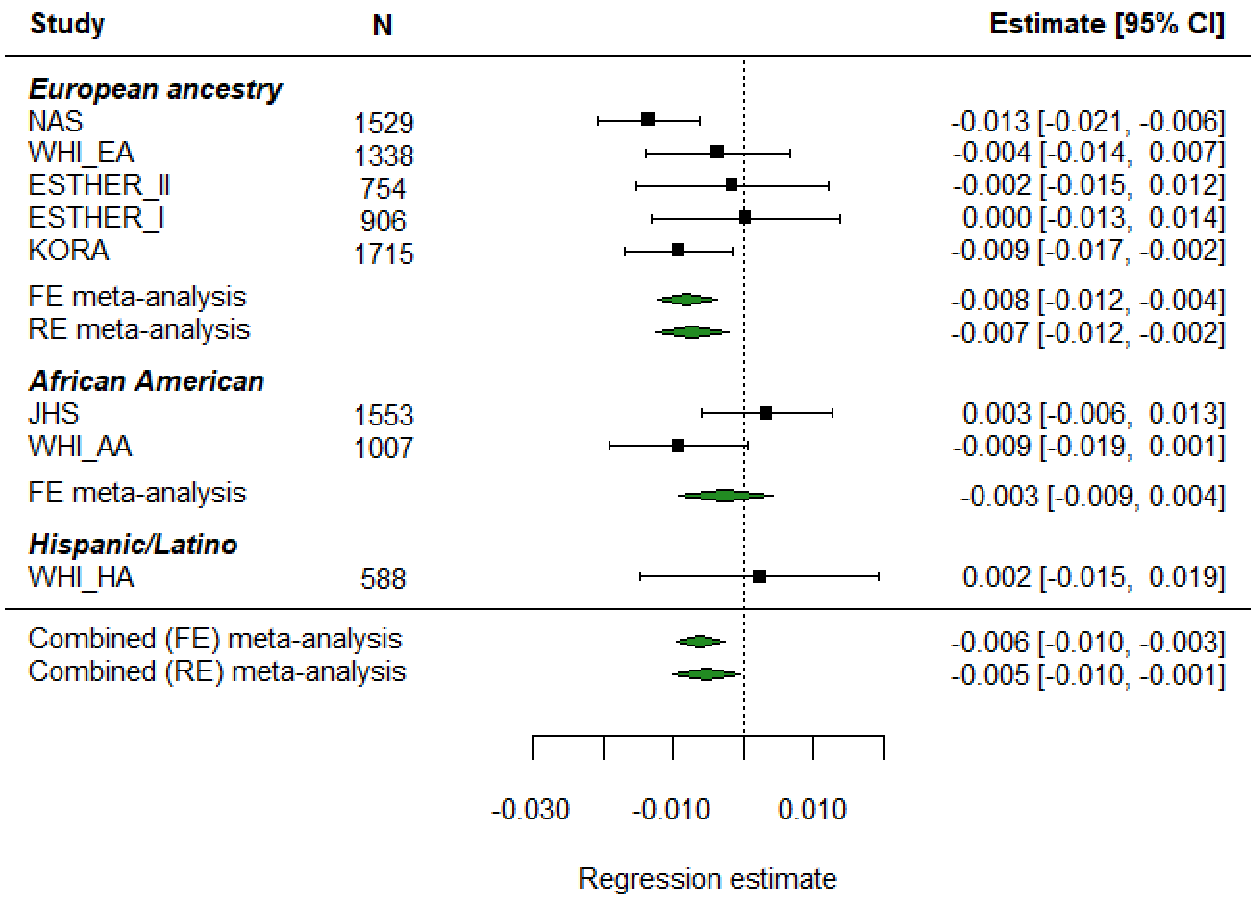

Suppl. Figure 3. Forest plots of associations with uACR

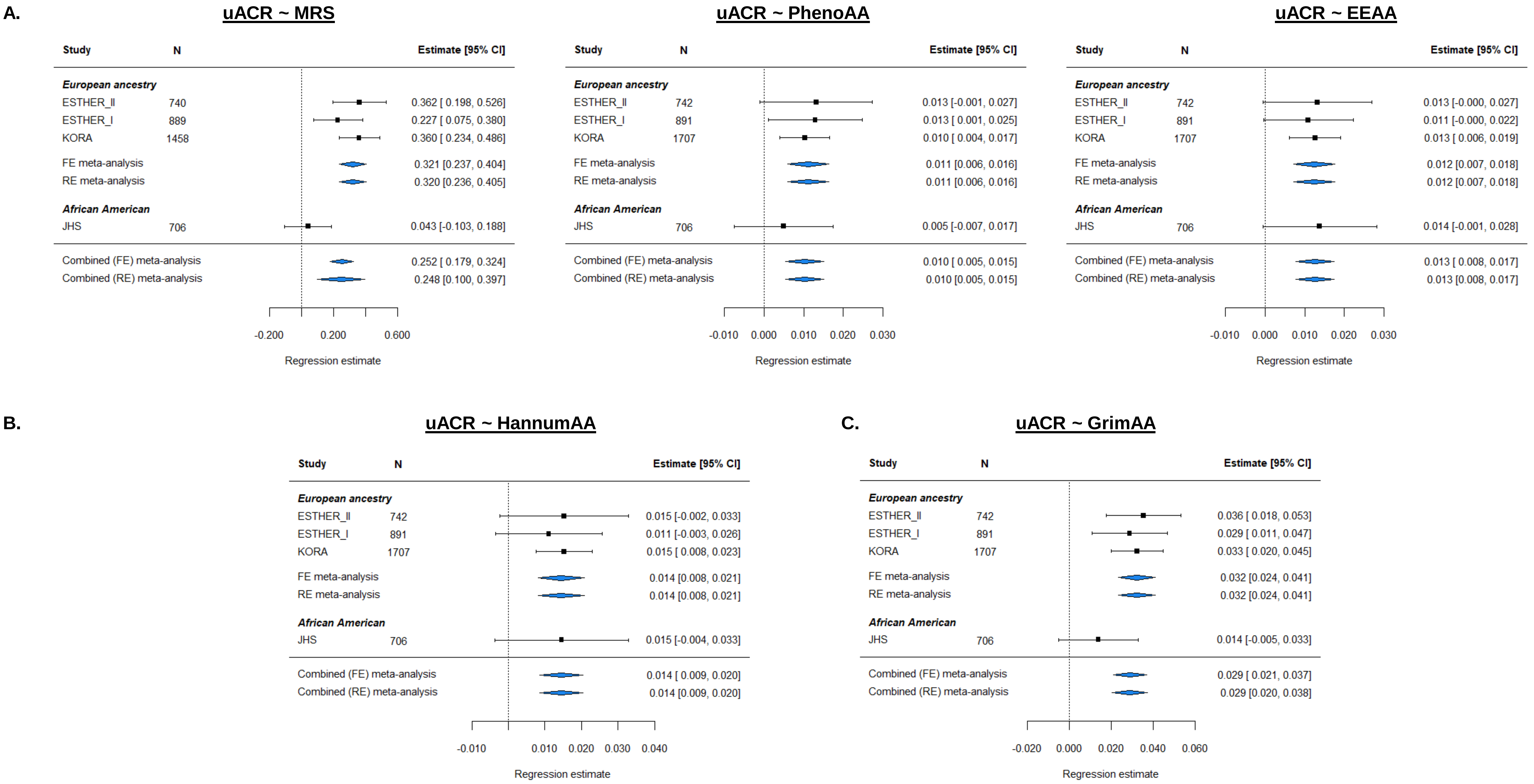

Suppl. Figure 4. Forest plots of associations with microalbuminuria

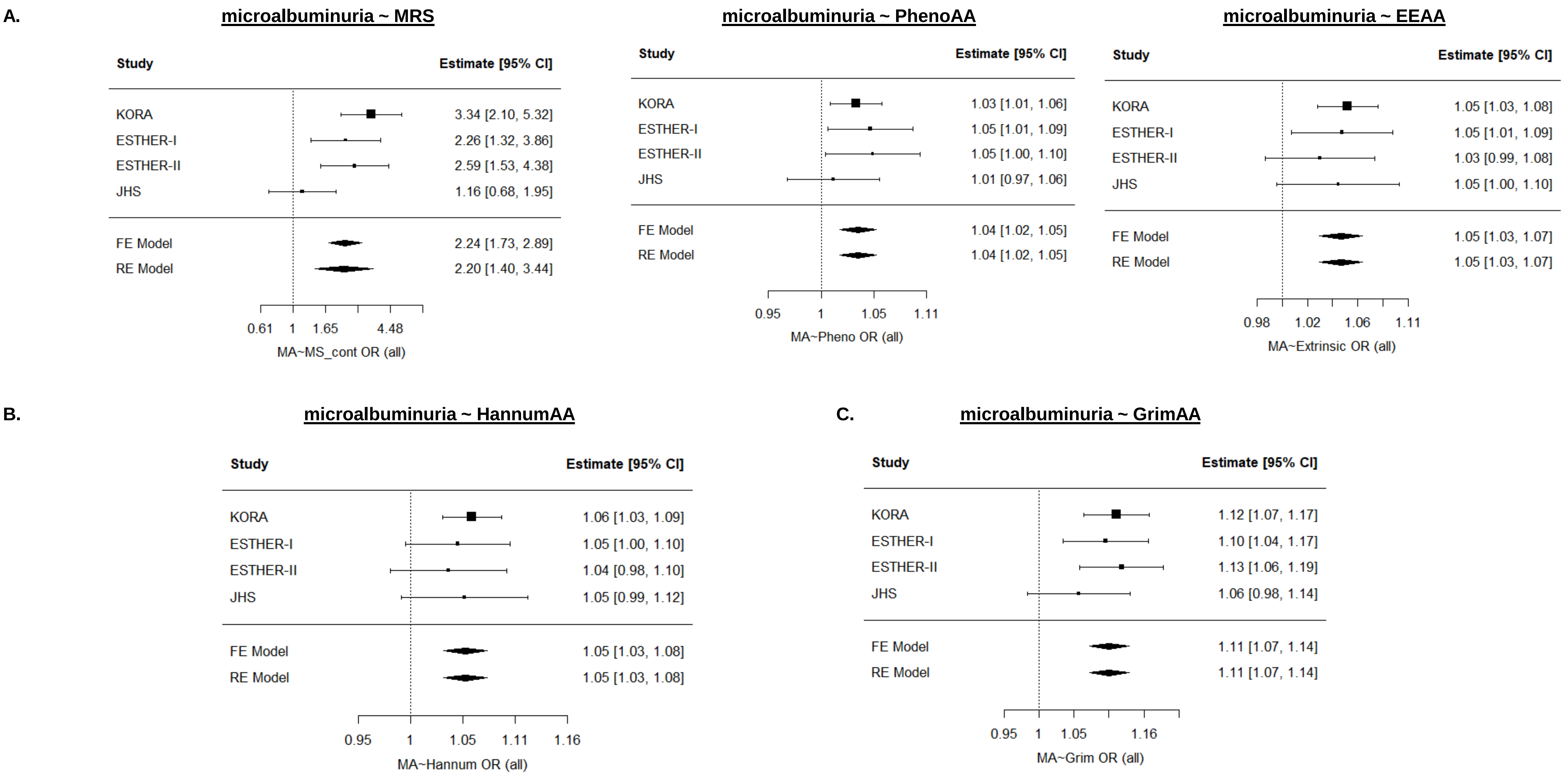

Suppl. Figure 5. Forest plots of associations with urate

A.

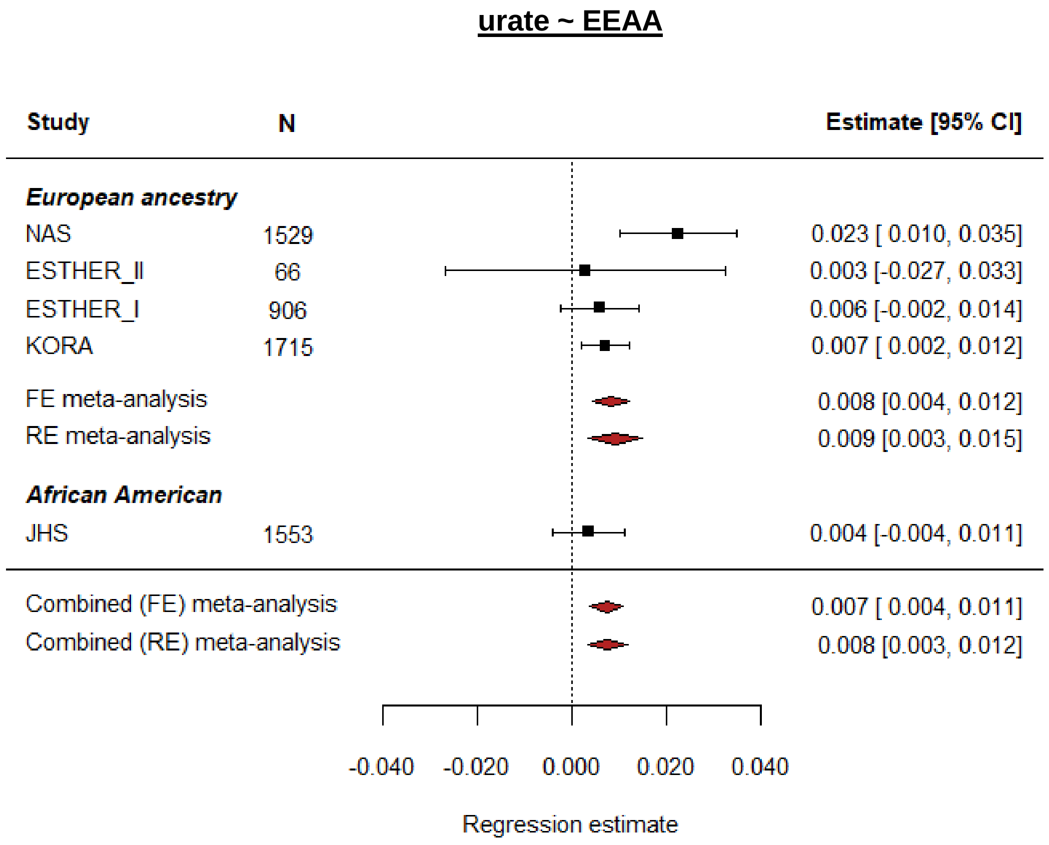

B.

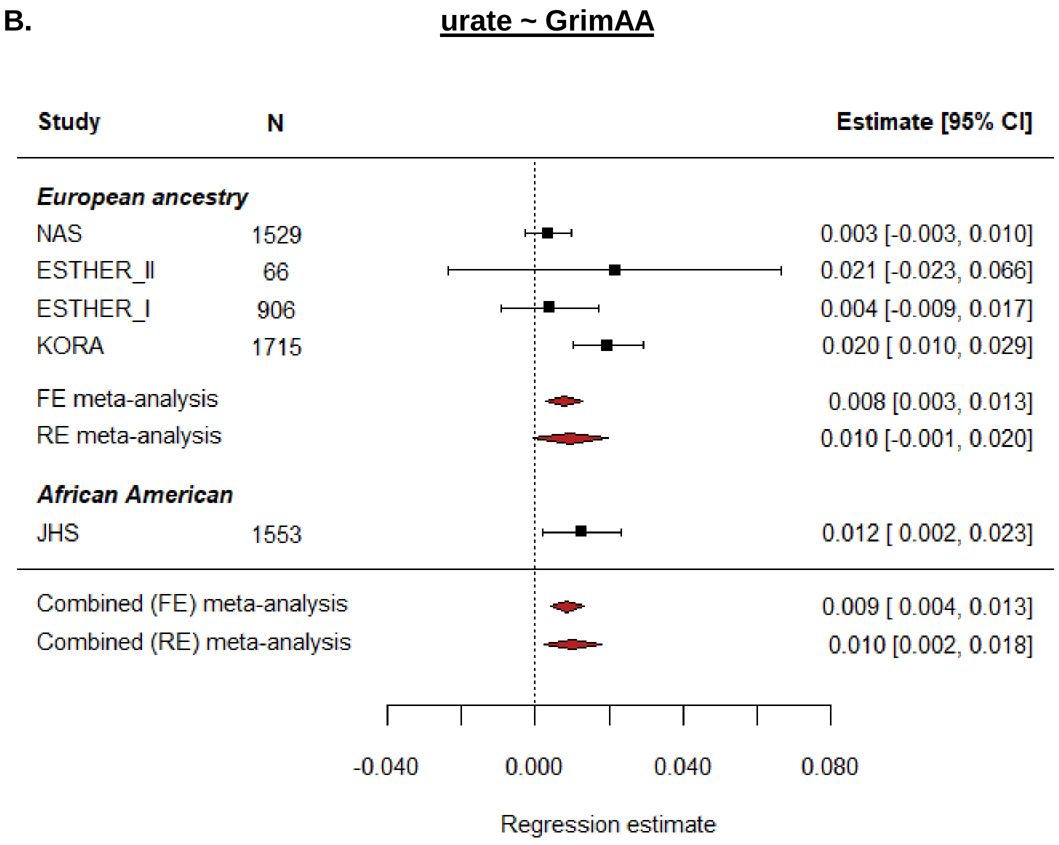

Suppl. Figure 6. Forest plots for secondary associations with uACR and serum urate

A.

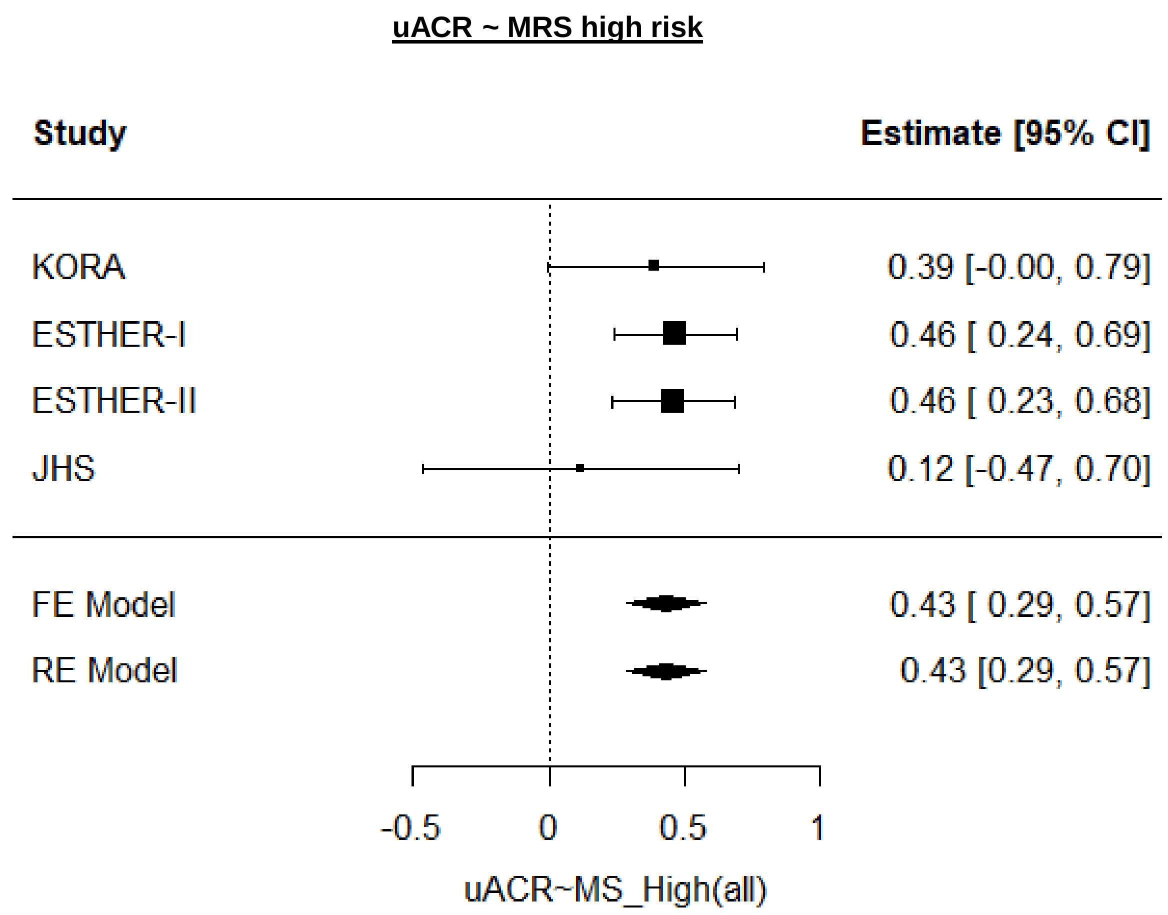

B.

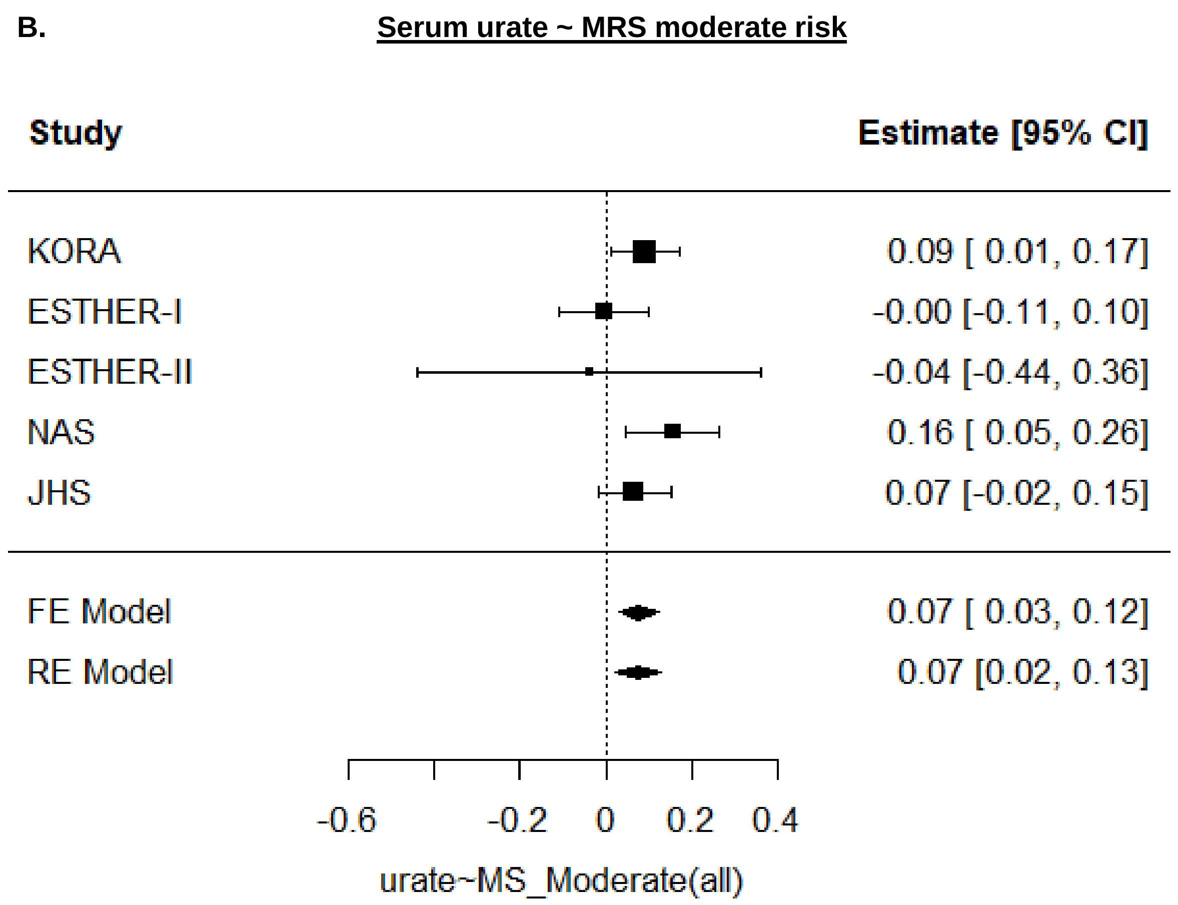

Suppl. Figure 7. Effect estimates from MRS and renal traits

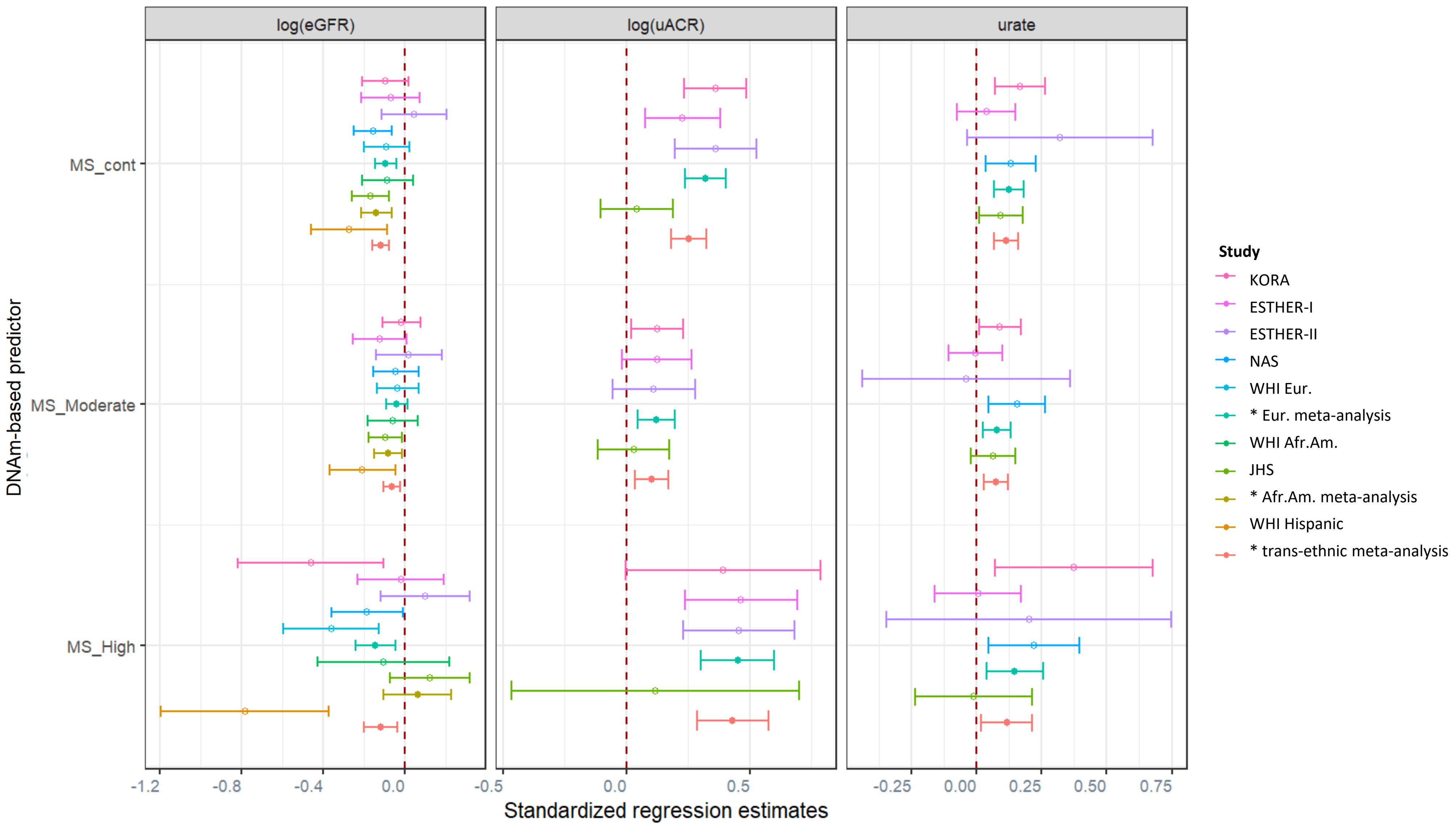

Supplement: Supplementary file 2 — Additional file 2: Figures S1–S7 showing forest plots for all significant associations not presented in the main figures, as well as secondary traits (categorical MRS), comparison of MRS and DNAmAA effect sizes across renal traits. [file 13148_2021_1082_MOESM2_ESM.pdf]
