# Supplementary material for: DNAm-based signatures of accelerated aging and mortality in blood are associated with low renal function
Source: Clin Epigenetics. 2021 Jun 2;13:121. doi: 10.1186/s13148-021-01082-w (PMC8170969; doi:10.1186/s13148-021-01082-w)
Supplement: Supplementary file 3 — Additional file 3: Notes including additional information on sensitivity analyses, the derivation of the DNAm-based predictors, cohort-specific details (background and study design, as well as data collection and pre-processing), and all legends to supplemental tables and figures. [file 13148_2021_1082_MOESM3_ESM.docx]

**Epigenetic measures of accelerated aging and mortality in blood are associated with low renal function**

**Additional file**

**Table of Contents**

[Legends to Supplemental Figures 2](#_Toc68554374)

[Additional file 2: Figure S1. Odds Ratios from DNAm-based predictors and binary kidney traits 2](#_Toc68554375)

[Additional file 2: Figure S2. Forest plots of associations with eGFR and CKD 2](#_Toc68554376)

[Additional file 2: Figures S3 and S4. Forest plots of associations with uACR and microalbuminuria 2](#_Toc68554377)

[Additional file 2: Figure S5. Forest plots of associations with urate 3](#_Toc68554378)

[Additional file 2: Figure S6. Forest plots for secondary associations with serum urate and uACR 3](#_Toc68554379)

[Additional file 2: Figure S7. Effect estimates from MRS and renal traits 4](#_Toc68554380)

[Legends to Supplemental Tables 4](#_Toc68554381)

[Additional file 1: Table S1. Population characteristics of participating studies, extended 4](#_Toc68554382)

[Additional file 1: Table S2. Study-level regression results for all kidney traits 4](#_Toc68554383)

[Additional file 1: Table S3. Results from meta-analyses of fully-adjusted model 5](#_Toc68554384)

[Additional file 1: Table S4. Standardized study-level and meta-analysis results 5](#_Toc68554385)

[Additional file 1: Table S5. Meta-analytic results for DNAm-estimated traits 6](#_Toc68554386)

[Supplemental Notes 7](#_Toc68554387)

Additional file 3: [Note S1. Sensitivity analyses 7](#_Toc68554388)

[***1.1 Multicollinearity and estimation of variance-inflation factor*** 7](#_Toc68554389)

[***1.2 Change-in-effect method*** 8](#_Toc68554390)

[Additional file 3: Note S2. DNAmAge predictors 15](#_Toc68554391)

[Additional file 3: Note S3. Additional cohort-specific information 16](#_Toc68554392)

[**KORA** 16](#_Toc68554393)

[**ESTHER** 18](#_Toc68554394)

[**NAS** 20](#_Toc68554395)

[**WHI** 22](#_Toc68554396)

[**JHS** 24](#_Toc68554397)

[References 27](#_Toc68554398)

# **Legends to Supplemental Figures**

## **Additional file 2: Figure S1. Odds Ratios from DNAm-based predictors and binary kidney traits**

Scatter plot showing the effect estimates from the DNAmAge and lifespan predictors across binary kidney traits for individual studies and trans-ethnic fixed-effects meta-analysis. The legend shows the combination of shape and color coding assigned for the studies.

## **Additional file 2: Figure S2. Forest plots of associations with eGFR and CKD**

Forest plot showing the standardized regression coefficients of 7 studies examining the association between measures of DNAmAge acceleration or changes in DNAm lifespan predictors and serum creatinine-based traits, namely estimated glomerular filtration rate (eGFR) and chronic kidney disease (CKD). The rows correspond to the different studies and the sample size for each analysis (N). For eGFR, the regression estimates represent the change in one standard deviation of the kidney trait per one year of age acceleration for PhenoAA and EEA. For CKD, the estimate column corresponds to the odds ratio (OR). The x-axis shows the estimates obtained from either the regression model (for single studies, data point shape is a black square) or the meta-analytic estimate (data point shape is a green diamond for serum creatinine-based traits) with their 95% CI. Panel A shows CKD-EEAA and eGFR-PhenoAA associations, which were statistically significant at the Bonferroni-corrected level in the trans-ethnic meta-analyses and nominally significant in either of the ethnic-specific meta-analyses. Panel B shows effects of both kidney traits for one year of Hannum age acceleration (HannumAA), and panel C shows the effect of one year of Horvath age acceleration (HorvathAA) for eGFR. KORA (European ancestry study), Kooperative Gesundheitsforschung in der Region Augsburg; ESTHER (European ancestry study), Epidemiologische Studie zu Chancen der Verhütung, Früherkennung und optimierten THerapie chronischer ERkrankungen in der älteren Bevölkerung; WHI, Women’s Health Initiative; EA, European ancestry; AA, African American; NAS (European ancestry study), Normative Aging Study; JHS (African American study), Jackson Heart Study.

## **Additional file 2: Figure S3 and S4. Forest plots of associations with uACR and microalbuminuria**

Forest plot showing the standardized regression coefficients of four studies examining the association between measures of DNAmAge acceleration or changes in DNAm lifespan predictors and urinary albumin-to-creatinine ratio (uACR) and microalbuminuria (uACR ≥ 30 mg/g). The rows correspond to the different studies and the sample size for each analysis (N). For uACR, the regression estimates represent the change in one standard deviation of the kidney trait per one year of age acceleration for PhenoAA and EEA and per unit increase in the continuous MRS. For microalbuminuria, the estimate column corresponds to the odds ratio (OR). The x-axis shows the estimates obtained from either the regression model (for single studies, data point shape is a black square) or the meta-analytic estimate (data point shape is a blue diamond) with their 95% CI. Panel A shows associations between these kidney traits and the three “universally” associated DNAm-based predictors. Panel B shows the associations with age acceleration measured by one of the two “first-generation” DNAm-based clocks, HannumAA, and panel C an analogous measure of age acceleration in the mortality predictor GrimAge (GrimAA). KORA, Kooperative Gesundheitsforschung in der Region Augsburg; ESTHER (European ancestry study), Epidemiologische Studie zu Chancen der Verhütung, Früherkennung und optimierten THerapie chronischer ERkrankungen in der älteren Bevölkerung; JHS (African American study), Jackson Heart Study. KORA (European ancestry study), Kooperative Gesundheitsforschung in der Region Augsburg; ESTHER (European ancestry study), Epidemiologische Studie zu Chancen der Verhütung, Früherkennung und optimierten THerapie chronischer ERkrankungen in der älteren Bevölkerung.

## **Additional file 2: Figure S5. Forest plots of associations with urate**

Forest plot showing the standardized regression coefficients of four studies examining the association between measures of DNAmAge acceleration or changes in DNAm lifespan predictors and serum urate. The rows correspond to the different studies and the sample size for each analysis (N). The regression estimates represent the change in one standard deviation of the kidney trait per one year of age acceleration for EEAA and GrimAge (GrimAA). The x-axis shows the estimates obtained from either the regression model (for single studies, data point shape is a black square) or the meta-analytic estimate (data point shape is a red diamond) with their 95% CI. Panel A shows associations between serum urate and EEAA. Panel B shows the association an analogous measure of age acceleration in the mortality predictor GrimAge (GrimAA). KORA, Kooperative Gesundheitsforschung in der Region Augsburg; NAS; Normative Aging Study; ESTHER, Epidemiologische Studie zu Chancen der Verhütung, Früherkennung und optimierten THerapie chronischer ERkrankungen in der älteren Bevölkerung; JHS, Jackson Heart Study.

## **Additional file 2: Figure S6. Forest plots for secondary associations with serum urate and uACR**

Forest plot showing the standardized regression coefficients of studies examining the association between serum urate, uACR and the categorical variables of the MRS. The figure shows the standardized regression coefficients obtained from linear regression models (with their corresponding 95% confidence intervals) in the individual studies, as well as those obtained from fixed- and random-effects combined-ethnicity meta-analyses. Panel A shows uACR estimates for the high MRS risk category and panel B shows the serum urate estimates for the high risk categorical variable of MRS. KORA, Kooperative Gesundheitsforschung in der Region Augsburg; NAS; Normative Aging Study; ESTHER, Epidemiologische Studie zu Chancen der Verhütung, Früherkennung und optimierten THerapie chronischer ERkrankungen in der älteren Bevölkerung; JHS, Jackson Heart Study.

## **Additional file 2: Figure S7. Effect estimates from MRS and renal traits**

Scatter plot showing the standardized effect estimates from the continuous and categorical MRS across continuous renal traits calculated in the individual studies and in meta-analyses. Studies are color coded, and fixed-effect meta-analyses results are marked with a star in the legend. MRS, epigenetic mortality risk score; MRS moderate, MRS risk category defined by 2-5 aberrantly methylated CpG sites; MRS high, MRS risk category defined >5 aberrantly methylated CpG sites. KORA, Kooperative Gesundheitsforschung in der Region Augsburg; ESTHER, Epidemiologische Studie zu Chancen der Verhütung, Früherkennung und optimierten THerapie chronischer ERkrankungen in der älteren Bevölkerung; NAS, Normative Aging Study; WHI Eur., Women’s Health Initiative (European ancestry cohort); Eur. meta-analysis, European-specific meta-analysis; WHI Afr.Am., Women’s Health Initiative (African American cohort); JHS, Jackson Heart Study; Afr.Am. meta-analysis, African American-specific meta-analysis; WHI Hispanic, Women’s Health Initiative (Hispanic ancestry cohort); trans-ethnic meta-analysis, final meta-analysis combining all studies.

# **Legends to Supplemental Tables**

## **Additional file 1: Table S1. Population characteristics of participating studies, extended**

Table showing additional population characteristics of all participating studies. The mean and (SD) is shown for continuous traits, as N and (%) for binary traits. Skewed variables are marked with **, for which median and (1st, 3rd quartile) are shown. Shown are the DNAm age acceleration (AA) measures, calculated as the difference between predicted DNAm age and chronological age, followed by DNAm-estimated traits used in the estimation of GrimAge. Additional information on blood cell composition, as well as measurement methods of serum creatinine and DNAm data, is included. ”\” denotes the trait was not available.

## **Additional file 1: Table S2. Study-level regression results for all kidney traits**

Table showing study-level regression estimates from both models assessing the association between all kidney traits and DNAmAge acceleration and changes in DNAm lifespan predictors. Regression coefficients from continuous variables (log-transformed eGFR, log-transformed uACR and serum urate) are standardized to their standard deviations, whereas no standardization was done to regression coefficients from binary traits (prevalent chronic kidney disease [CKD] and microalbuminuria). Model 1 was a basic model with adjustment for chronological age and sex, and model 2 additionally included BMI, log transformed triglycerides, HDL, hypertension, smoking status and diabetes. Estimate, regression coefficient; SE, standard error; CI lower, lower bound of 95%CI of regression coefficient; CI upper, upper bound of 95%CI of regression coefficient; OR, odds ratio; OR CI lower, lower bound of 95%CI of OR; CI upper, upper bound of 95%CI of OR; P, pvalue; N, sample size.

## **Additional file 1: Table S3. Results from meta-analyses of fully-adjusted model**

Table showing results obtained from fixed-effects (inverse variance-weighted) and random-effects (Restricted Maximum-Likelihood estimator) meta-analyses of the fully-adjusted regression coefficients from participating studies (model 2). The column “group” shows: all, combined-ethnicity analyses; EA, European ancestry analyses; AA, African American analyses. beta, estimated coefficients of the model; se, standard errors of the coefficients; zval, test statistics of the coefficients; pval, p-values for the test statistics; ci.lb, lower bound of 95% confidence interval for the coefficient; ci.ub, upper bound of 95% confidence interval for the coefficient; OR, odds ratio calculated from regression coefficient; OR.lb, lower bound of 95% confidence interval for the OR; OR.ub, upper bound of 95% confidence interval for the OR; vb, variance-covariance matrix of the estimated coefficients; k, total number of included studies; QE, test statistic for the test of (residual) heterogeneity; QEp, p-value for the test of (residual) heterogeneity; QM, test statistic for the omnibus test of coefficients; QMp, p-value for the omnibus test of coefficients; I2, I^2^ value estimating the proportion of the total variability in the effect size estimates attributable to heterogeneity; H2, H^2^ value estimating ratio of the total amount of variability in the effect size estimates to the amount of sampling variability; FE, fixed-effects meta-analysis; RE, random-effects meta-analysis. Additional columns specific to random-effects meta-analysis: tau2, or tau^2^, variance of the distribution of true effect sizes (estimated amount of residual heterogeneity); se.tau2, estimated standard error of the estimated amount of (residual) heterogeneity.

## **Additional file 1: Table S4.** **Standardized study-level and meta-analysis results**

Table showing standardized study-level and meta-analytic regression estimates from the fully adjusted models investigating the association between continuous kidney traits (log-transformed eGFR, log-transformed uACR and serum urate) and age acceleration in the six main DNAm predictors (HannumAA, HorvathAA, EEAA, PhenoAA, GrimAA and MRS).

The fully adjusted model included chronological age, sex, BMI, log transformed triglycerides, HDL, hypertension, smoking status and diabetes. Linear regression coefficients were standardized to one SD in the exposure and outcome variables, using the reported SD information from Table 1 and Additional file 1: Table S1. Estimate.xy, standardized regression coefficient; SE.xy, standard error of the standardized regression coefficient; CI lower, lower bound of 95%CI of standardized regression coefficient; CI upper, upper bound of 95%CI of standardized regression coefficient; P, pvalue; study: name of study or fixed-effects meta-analysis (MA_FE). The column “study” shows: all, combined-ethnicity analyses; EA, European ancestry analyses; AA, African American analyses.

## **Additional file 1: Table S5. Meta-analytic results for DNAm-estimated traits**

Table showing results obtained from fixed-effects (inverse variance-weighted) and random-effects (Restricted Maximum-Likelihood estimator) meta-analysis. The column “group” shows: all, combined-ethnicity analyses; EA, European ancestry analyses; AA, African American analyses. beta, estimated coefficients of the model; se, standard errors of the coefficients; zval, test statistics of the coefficients; pval, p-values for the test statistics; ci.lb, lower bound of 95% confidence interval for the coefficient; ci.ub, upper bound of 95% confidence interval for the coefficient; vb, variance-covariance matrix of the estimated coefficients; k, total number of included studies;QE, test statistic for the test of (residual) heterogeneity; QEp, p-value for the test of (residual) heterogeneity; QM, test statistic for the omnibus test of coefficients; QMp, p-value for the omnibus test of coefficients;

I2, I^2^ value estimating the proportion of the total variability in the effect size estimates attributable to heterogeneity; H2, H^2^ value estimating ratio of the total amount of variability in the effect size estimates to the amount of sampling variability; FE, fixed-effects meta-analysis; RE, random-effects meta-analysis.Additional columns specific to random-effects meta-analysis: tau2, or tau^2^, variance of the distribution of true effect sizes (estimated amount of residual heterogeneity); se.tau2, estimated standard error of the estimated amount of (residual) heterogeneity.

# **Additional file 3: Notes**

## **Additional file 3: Note S1. Sensitivity analyses**

Sensitivity analyses were conducted in KORA F4 (N = 1,725), due to it being the largest contributing cohort to this study with available data on all investigated DNAm-based predictors, kidney traits and potential confounding variables.

### ***1.1 Multicollinearity and estimation of variance-inflation factor***

The interpretation of the regression coefficients will greatly depend/vary if the linear regression model features correlated independent variables. Multicollinearity, occurring when two or more independent variables are correlated, can be detected calculating the variance inflation factor (VIF). VIF for the x^th^ independent variable is calculated as:

${VIF}_{x}= \frac{1}{1-R_{x}^{2}}$

where$R_{x}^{2}$ corresponds to the R^2^-value obtained by regressing the x^th^ independent variable on the remaining variables. In general, if VIF = 1 means there is no correlation among the x^th^ variable and the remaining variables (thus the variance of bx is not inflated), whereas VIFs exceeding 4 require further investigation and those >10 point to strong multicollinearity. The *vif()* function from the *car* library was used calculate the VIF for each independent variable in the fully-adjusted models. As an example, the following plot shows the VIF for each independent variable included in the regression model with log-transformed uACR as outcome and the epigenetic mortality risk score (MRS) as predictor:

log-transformed uACR ~ MRS + age + sex + smoking + log-transformed triglycerides + hypertension + HDL + diabetes + BMI


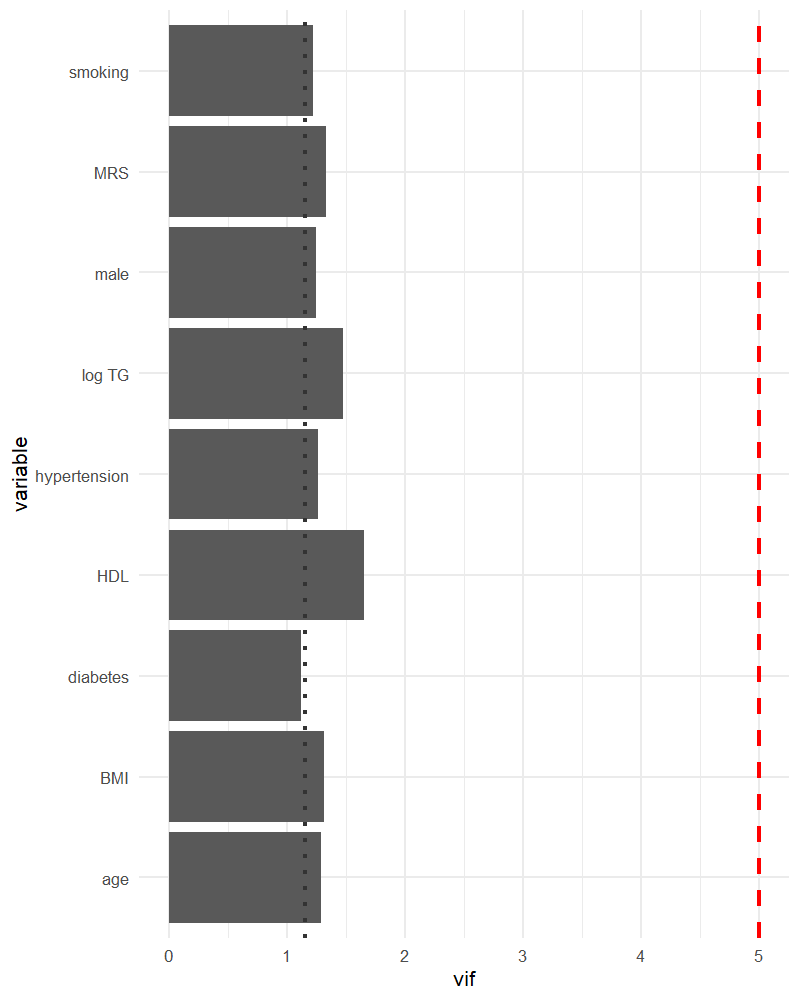


**Additional file 3: Note Fig. S1. Variance-inflation factor plot for all variables included in the uACR-MRS fully-adjusted model**

As can be seen in this plot, no multicollinearity was identified in the set of included variables (VIF < 5 for all variables, threshold for multicollinearity as dotted red line). Similar observations were done for other kidney traits and DNAm-based predictors. This allows for the interpretation of effect changes as a viable approach to identify confounding and mediating factors, presented in the next section.

### ***1.2 Change-in-effect method***

The change-in-effect estimate method, an approach calculating the changes in effect estimates derived from the addition of a new variable to a regression model, was applied to the set of variables included in the fully-adjusted model from the main analysis. This method consists of the introduction of variables to the model in a step-wise manner, starting with a basic model and sequentially adding the variable with the largest change from the set of eligible variables (PMID: 25785886, doi:10.1146/annurev-publhealth-031914-122559). It has been shown to produce more reliable models than variable selection methods based on statistical significance (https://doi.org/10.1201/b16851). The R packages *chest* v.0.3.5 and *foresplot* v.1.10.1 were used in these analyses (doi:10.1177/1536867X0700700203 and <https://gforge.se/packages/> ).

As a first step, the basic model was defined as the “crude” bivariate association between the DNAm-based predictor and the kidney trait:

y ~ β_0_ + β_1_*DNAm predictor

And added variables to this model in a step-wise manner, where the variable “X_1_” was added in step 1:

y ~ β_0_ + β_1_*DNAm predictor + β_x1_*X_1_

The change-in-estimate is defined as the change in the regression coefficient of the main predictor (DNAm-based predictor in the regression models) caused by introducing a variable X to the model, and is expressed as the “relative” change in β_1_ = (β_1, basic model_ - β_1, augmented model_)/β_1, basic model_×100%.

To follow-up on the example presented in section 1.1, the plots below shows the change-in-estimate for the association between log-transformed uACR and MRS, with the basic model defined as the “crude” model:

log-transformed uACR ~ MRS


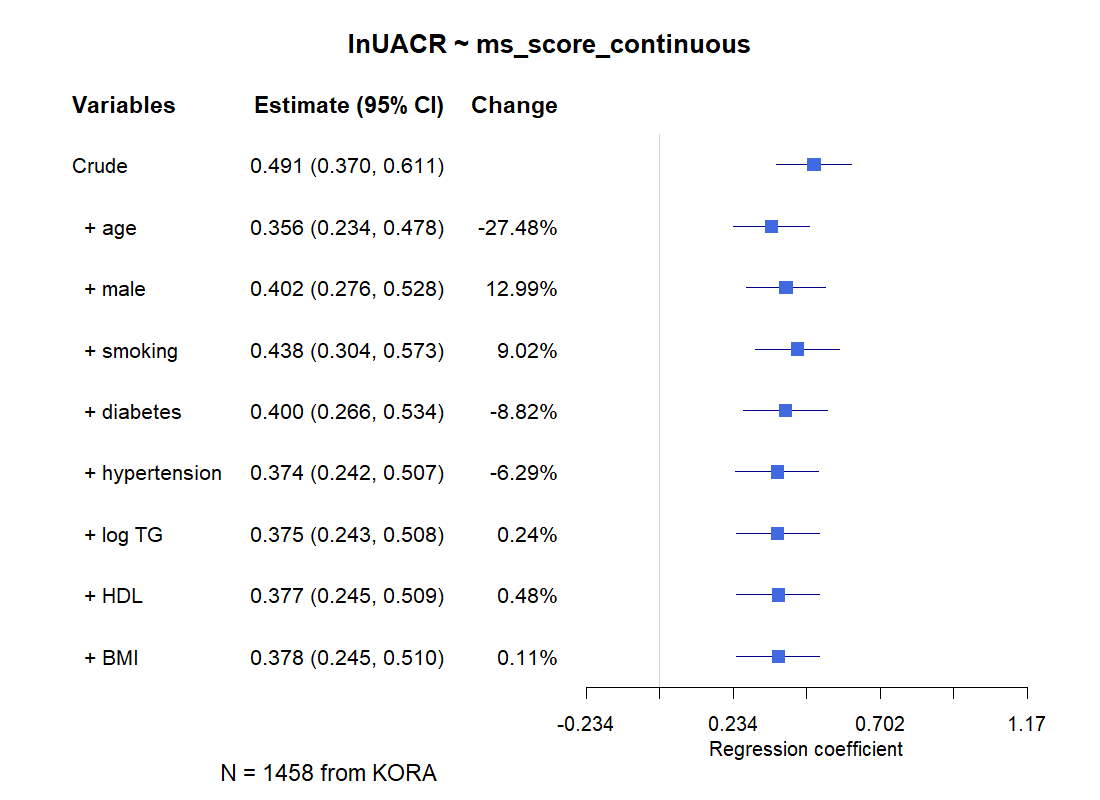


**Additional file 3: Note Fig. S2. Change-in-effect plot for variables included in the uACR-MRS “crude” model**

As can be seen in highlighted with an orange rectangle **Additional file 3: Note Fig. S2**, the coefficient for the association between uACR and MRS was attenuated by 27.48% after including chronological age in the “crude” model, followed by sex as the next variable causing the largest change-in-estimate (12.99%). The addition of other variables did not further alter the observed effect (highlighted with a green rectangle), despite a slight increase in the coefficient after the introduction of smoking (9.02% change in comparison to the age- and sex-adjusted coefficient).

Another example is the association between MRS and log-transformed eGFR: the largest changes-in-effect were observed again after the introduction of chronological aging to the “crude” model, where the “crude” effect (i.e. bivariate correlation) was attenuated by 82.49%. The introduction of the smoking variable had a 79.96% positive change-in-effect, although the effect remained largely unchanged therefrom (**Additional file 3: Note Fig. S3,** left plot). No evidence of strong collinearity between the DNAm-based predictors and other variables included was detected (**Additional file 3: Note Fig. S3,** right plot).


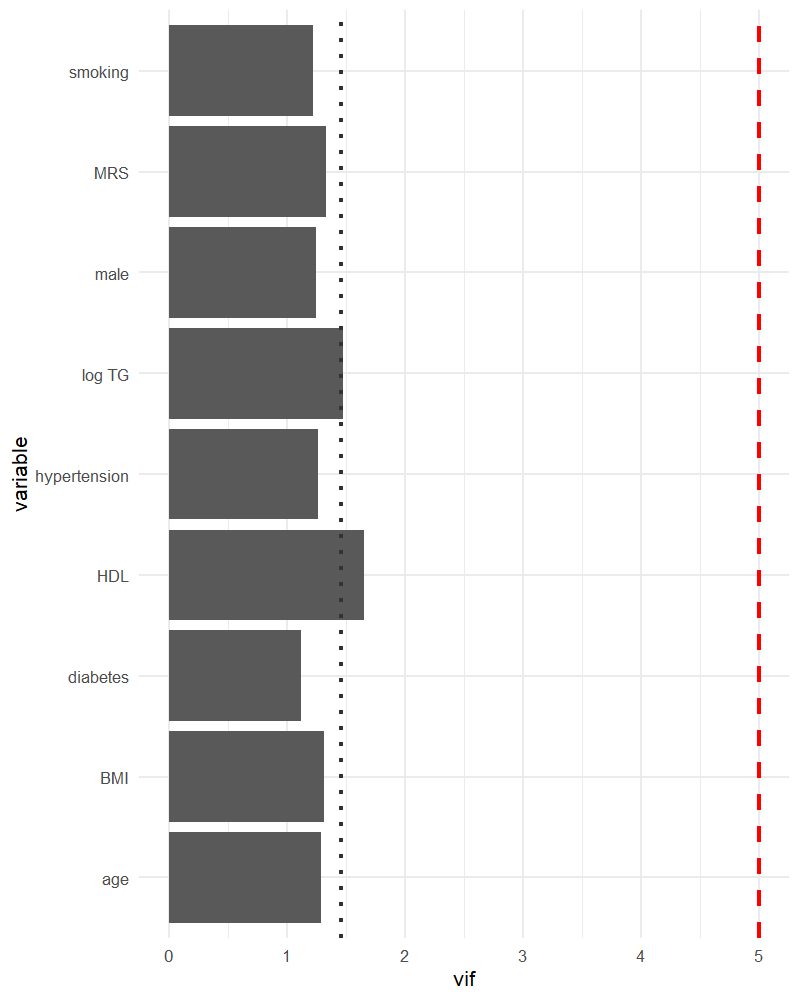

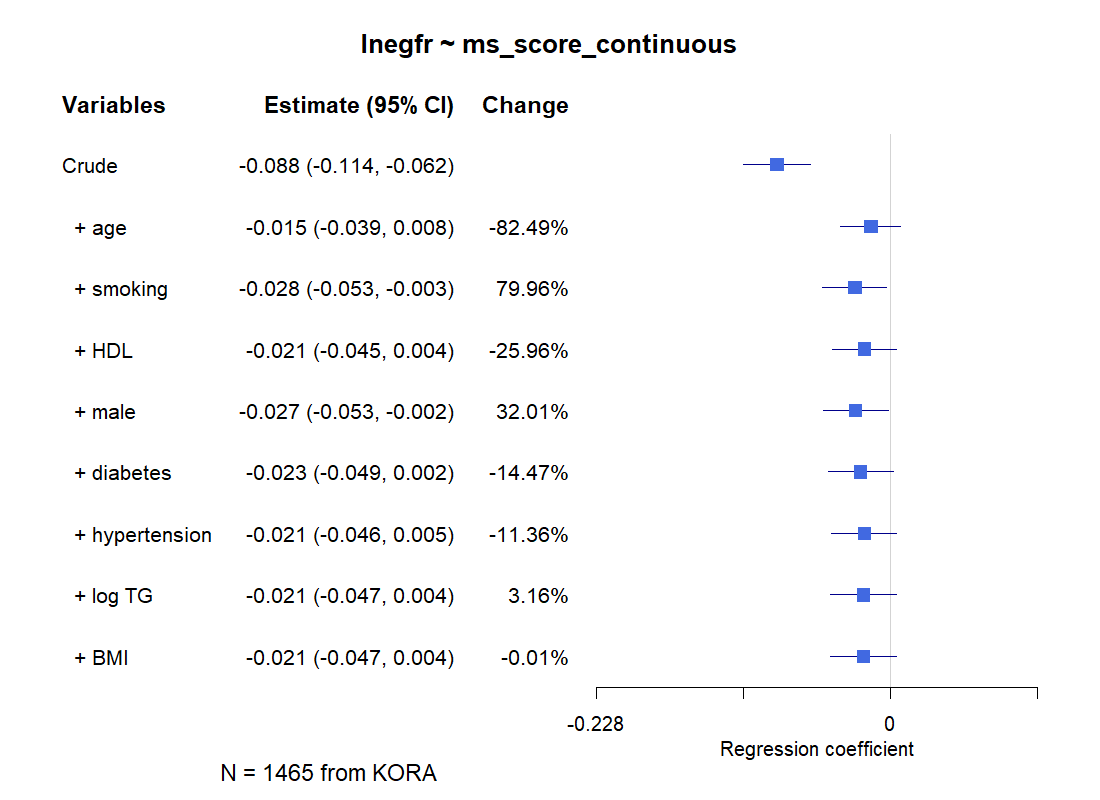


**Additional file 3: Note Fig. S3. Change-in-effect (top) and VIF plots (bottom) for eGFR-MRS fully-adjusted models**

Change-in-effect plots all from age- and sex-adjusted DNA-based predictor and kidney trait associations are shown in the next pages. Similar results were observed for the other sets of associations, where again the introduction of additional covariates to model 1 (adjusted for chronological age and sex) did not further largely alter the observed effect.


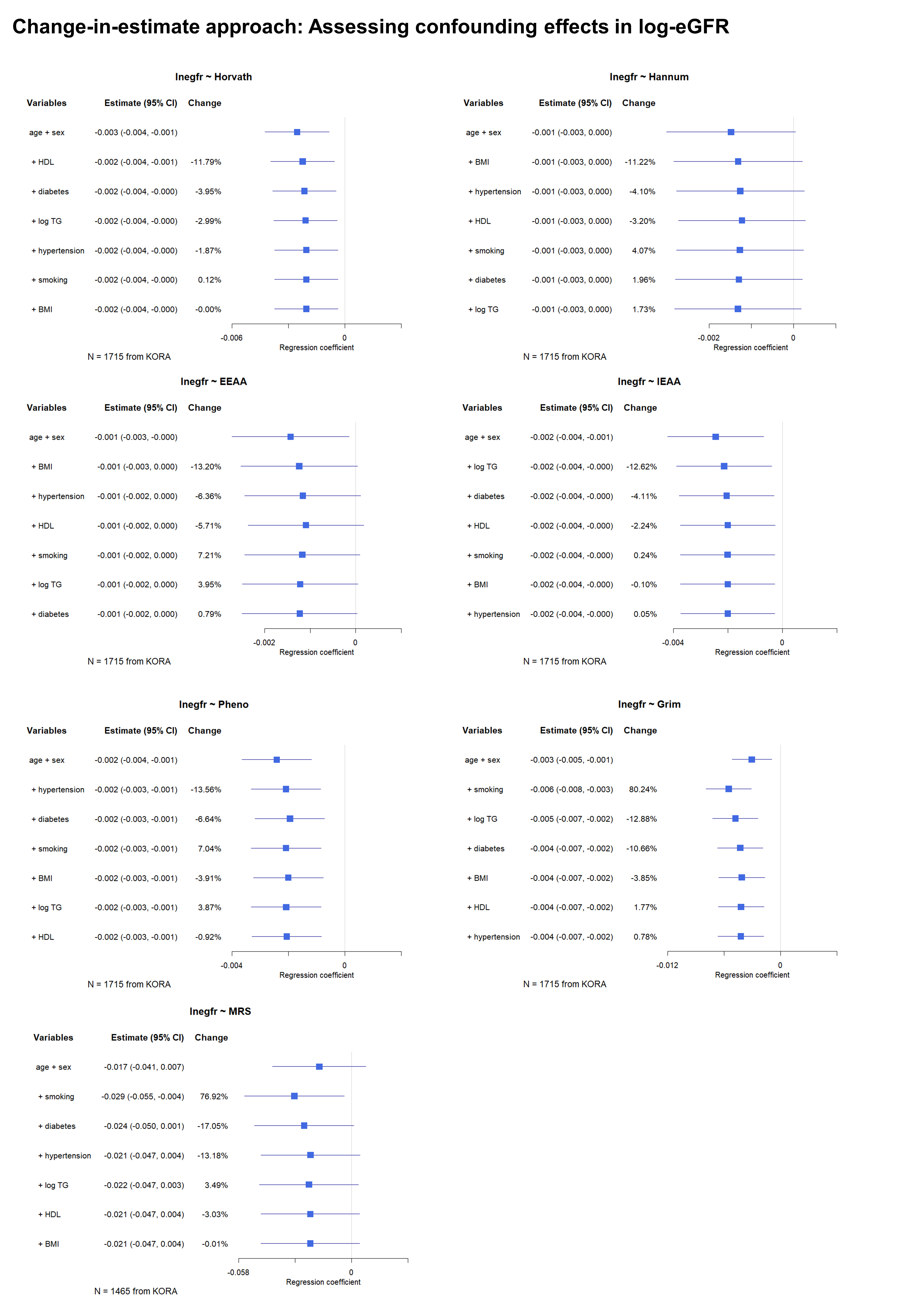

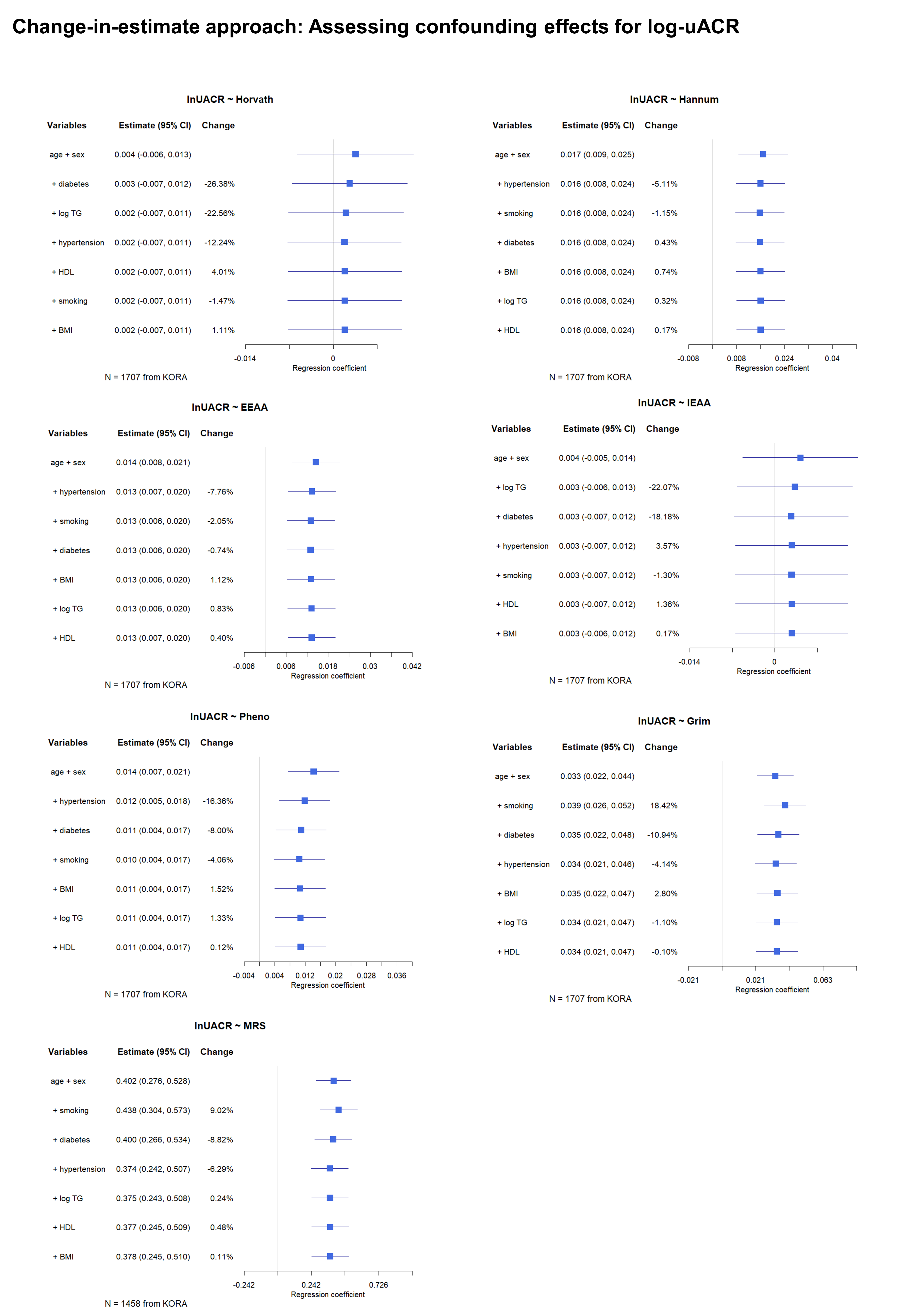

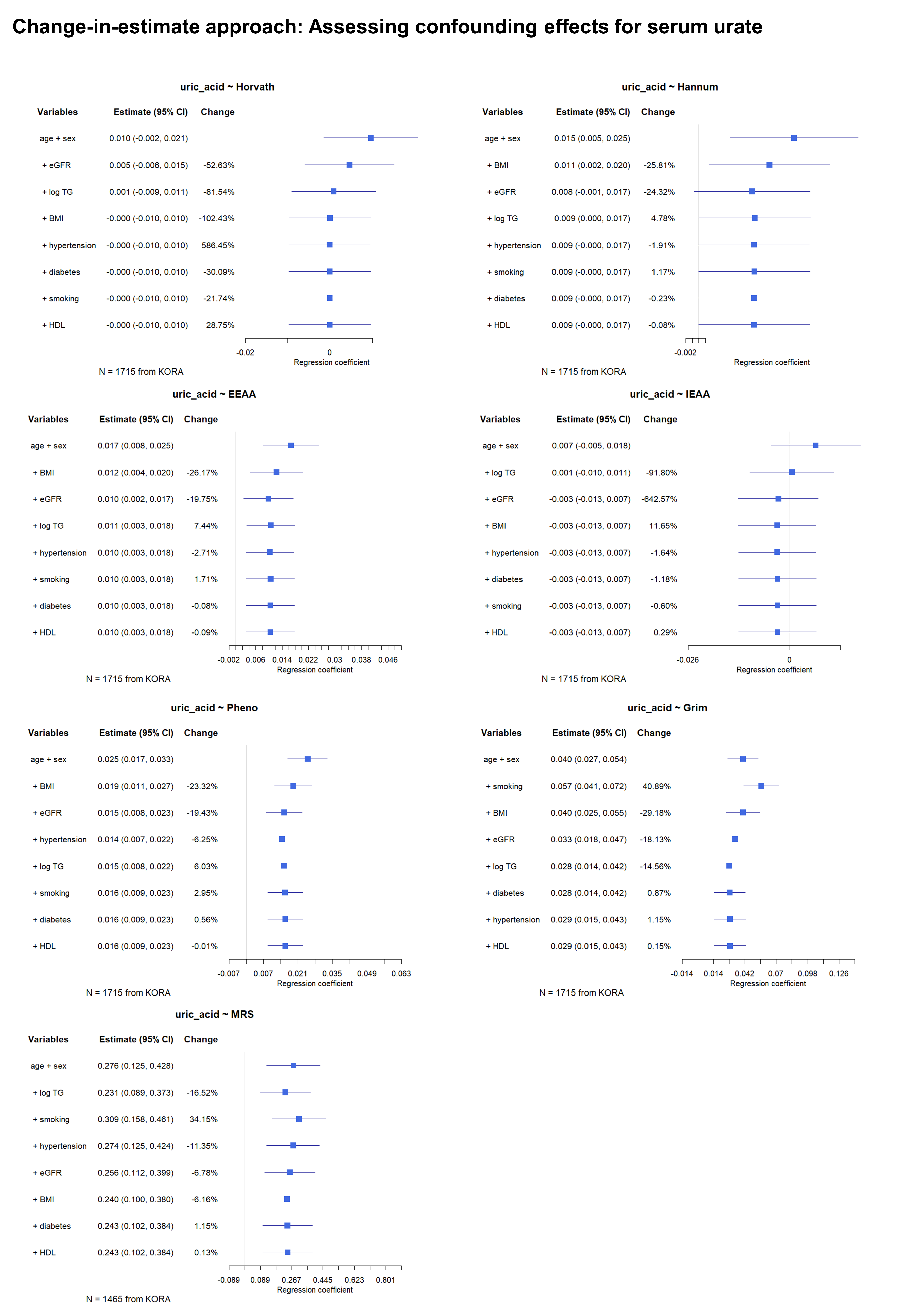


## **Additional file 3: Note S2. DNAmAge predictors**

HannumAge is a blood-specific estimate based on 71 CpG sites ^1^. ExtrinsicAge is derived from regressing HannumAge and three blood cell components (naïve cytotoxic T cells, exhausted cytotoxic T cells, and plasmablasts) on chronological age, thus capturing both DNAm age and age-related changes in blood cell composition. HorvathAge is a tissue-agnostic estimate based on 353 CpGs ^2^. PhenoAge is a 513 CpG-based biomarker trained on many aging-related clinical phenotypes (albumin, creatinine, glucose, C-reactive protein, alkaline phosphatase, white blood cell count) ^3^. GrimAge, based on 1030 CpG sites in total, uses seven DNAm-estimated plasma proteins (adrenomedullin (ADM), beta-2 microglobulin (B2M), growth differentiation factor 15 (GDF15), cystatin C, leptin, plasminogen activation inhibitor 1 (PAI1), tissue inhibitor metalloproteinase 1 (TIMP1)), and smoking (amount of cigarettes smoked, pack years) to produce its estimate ^4^.

## **Additional file 3: Note S3. Additional cohort-specific information**

### **KORA**

#### Cohort description

The KORA (Kooperative Gesundheitsforschung in der Region Augsburg) research platform has been collecting clinical and genetic data from individuals of German nationality from the general population living in the region of Augsburg in southern Germany for over 20 years. F4 (2006-2008) and FF4 (2013-2014) cohorts are follow-up studies from the KORA S4 (n=4,261) survey carried out 1999-2000. In the baseline examinations all inhabitants of German nationality between the ages of 25 and 74 years were enrolled. Participants completed a lifestyle questionnaire, including details on health status and medication use, underwent standardized examinations with blood samples taken; study design, sampling method and data collection have been described in detail elsewhere ^5^. The present study is based on a subsample of 1,727 participants of KORA F4 and with methylation and genotyping data available.

#### Ethics

The KORA cohort ethical approval was granted by the ethics committee of the Bavarian Medical Association (REC reference numbers: F4: #06068) and all were carried out in accordance with the principles of the Declaration of Helsinki. All research participants have signed informed consent prior to taking part in any research activities. The KORA data protection procedures were approved by the responsible data protection officer of the Helmholtz Zentrum München.

#### DNA Methylation

Genome-wide DNA methylation measurement at 485,577 genomic sites for KORA F4 was performed using the Infinium HumanMethylation450K BeadChip® (Illumina, Inc., CA, USA) ^6^ in 1802 KORA F4 samples. Sample preparation and measurement have been described previously ^7^. Methylated and unmethylated signal intensities were obtained for each CpG site, which were then converted to β-values, the ratio of the methylated signal intensity divided by the overall signal intensity ^6, 8^. DNA methylation data were preprocessed following the CPACOR pipeline of Lehne et al. ^9^. First, 65 probes that represent SNPs were excluded. Second, background correction was performed using the R package minfi, version 1.6.0 ^10^. Third, detection p-values were defined as the probability of a signal being detected above the background signal level, as estimated from negative control probes. Consequently, signals with detection p-values ≥ 0.01 were removed, as they indicate putatively unreliable signals. Similarly, signals summarized from less than three functional beads on the chip were characterized as potentially unreliable and removed from the data set. Observations with less than 95% of CpG sites providing reliable signals (75 in KORA F4) were excluded, resulting in 1727 samples overall.

Data were normalized using quantile normalization on the raw signal intensities. Beta-mixture quantile normalization was performed on a stratification of the probe categories into 6 types, based on probe type and color channel, using the R package limma, version 3.16.5 ^11^ . These data were then uploaded to Horvath’s online calculator, as described in the main text, to generate the various clock estimates.

#### Acknowledgements

The KORA study was initiated and financed by the Helmholtz Zentrum München – German Research Center for Environmental Health, which is funded by the German Federal Ministry of Education and Research (BMBF) and by the State of Bavaria. Furthermore, KORA research has been supported within the Munich Center of Health Sciences (MC-Health), Ludwig-Maximilians-Universität, as part of LMUinnovativ.

#### Data Availability

The informed consents given by KORA study participants do not cover data posting in public databases. However, data are available upon request from KORA Project Application Self-Service Tool (https://epi.helmholtz-muenchen.de/) Data requests can be submitted online and are subject to approval by the KORA Board.

### **ESTHER**

#### Cohort description

The ESTHER study is an ongoing population-based cohort study conducted in the federal state of Saarland, Germany ^12^. In brief, 9,949 older adults (50-75 years) were recruited by their general practitioners (GPs) during routine health check-ups (offered every two years to people older than 35 years in the German healthcare system) between 2000 and 2002, and followed up thereafter. During the baseline enrolment, epidemiological data (including socio-demographic characteristics, lifestyle factors, and history of major diseases) were collected via a standardized self-administered questionnaire completed by participants and via additional reports from participants’ GPs, and biological samples (blood, stool, urine) were obtained and stored at −80 °C. Two subsets of the ESTHER participants were selected for DNA methylation assessment in the baseline blood samples: Subset I consists of 1,000 participants consecutively enrolled during the first 3 months of recruitment; Subset_II consists of 864 participants selected for a case-cohort design for mortality analysis ^13^. Participants from the subset I and II with both methylation data and kidney function data available were included in the current study.

#### Ethics

The study was approved by the ethics committees of the University of Heidelberg and of the Medical Association of Saarland. All participants provided written informed consent.

#### DNA Methylation

DNA methylation in whole blood was quantified using the Infinium HumanMethylation450K BeadChip (Illumina.Inc, San Diego, CA, USA). In brief, 1.5 mg DNA (allocated in 96-well format with three random duplicate samples in each format as quality controls) was bisulfite converted, and 200 ng bisulfite-treated DNA was applied to the 450K BeadChips following the manufacturer’s instruction. Raw data pre-processing and initial quality control was carried out following the CPACOR pipeline ^9^. Probes with detection p-value>0.01 and targeting the sex chromosomes, cross-reactive probes, and polymorphic CpGs^14^ were removed before quantile normalization, which was applied following stratification of the probe type into 6 categories according to probe type and colour channel, using the R package limma^11^. Sample call rate threshold and CpG call rate threshold both were 95%. Epigenetic clock estimates were calculated using the online tool available at <https://dnamage.genetics.ucla.edu/>.

#### Regression analyses

No adjustment for HDL was done in the ESTHER-I and –II analyses due to >50% missingness in this variable.

#### Acknowledgements

The ESTHER study was supported by the Baden-Württemberg State Ministry of Science, Research and Arts (Stuttgart, Germany), the Federal Ministry of Education and Research (Berlin, Germany), and the Federal Ministry of Family Affairs, Senior Citizens, Women and Youth (Berlin, Germany). The sponsors had no role in the study design, in the collection, analysis, and interpretation of data and preparation, review, or approval of the manuscript.

### **NAS**

#### Cohort description

The NAS (Normative Aging Study) is an ongoing longitudinal study on aging established by the U.S. Department of Veterans Affairs in 1963. Details of the study have been published previously ^15^. Briefly, the NAS is a closed cohort of 2,280 male veterans from the Greater Boston area. They were enrolled after an initial health screening to determine that they were free of known chronic medical conditions. DNA from blood samples were collected from 1529 visits of 774 white male participants, whom were examined up to four times between 1999 and 2013. Participants have been reevaluated every three to five years on a continuous rolling basis using detailed on-site physical examinations and questionnaires.

As previously described ^15, 16^, participants were asked to provide detailed information about their lifestyles, dietary habits, activity levels, and demographic factors at each visit. Height and weight were used to calculate body mass index (BMI, in kg/m^2^). Blood samples were collected at each visit after overnight fasting to assess clinical biomarkers, such as total cholesterol (mg/dL), serum triglyceride (mg/dL), and high-density lipoprotein (HDL, mg/dL). Serum creatinine concentration (mg/dL) for estimating renal function was determined using Hitachi 747 analyzer (Boehringer-Mannheim Corp, Indianapolis, IN) . Systolic and diastolic blood pressures (SBP & DBP) were measured once in each arm while the subject was seated, using a standard cuff. Major diseases were assessed based on participants’ medical history and prior diagnoses.

#### Ethics

The NAS was approved by the Department of Veterans Affairs Boston Healthcare System and written informed consent was obtained from each subject before participation.

#### DNA Methylation

As previously described ^16^, we used the QIAamp DNA Blood Kit (Qiagen, CA, USA) to extract DNA from buffy coat and performed bisulfite conversion with the EZ-96 DNA Methylation Kit (Zymo Research, CA, USA). To minimize batch effects, we randomized chips across plates and randomized samples based on a two-stage age-stratified algorithm so that age distributed similarly across chips and plates. We measured DNA methylation of CpG probes using the Illumina HumanMethylation450 BeadChip. After quality control, the remaining samples were preprocessed using the Illumina-type background correction, dye-bias adjustment and BMIQ normalization to generate methylation status. The methylation status of a specific CpG site was quantified as a β value ranging from 0 (no methylation) to 1 (full methylation).

#### Regression analyses

Linear mixed models were used in this study to account for the correlation of repeated measures, where the participants’ identification number was included as a random effects term.

#### Acknowledgements

The Normative Aging Study is supported by the National Institute of Environmental Health Sciences (grants P30ES009089, R01ES021733, R01ES025225, and R01ES027747). The VA Normative Aging Study is supported by the Cooperative Studies Program/Epidemiology Research and Information Center of the U.S. Department of Veterans Affairs and is a component of the Massachusetts Veterans Epidemiology Research and Information Center, Boston, Massachusetts.

### **WHI**

#### Cohort description

WHI (Women’s Health Initiative) is a study of postmenopausal women (aged 50-79 years), comprising 161,808 women recruited from 40 U.S. clinical centers who participated in an observational study or in clinical trials during 1993-1998 as previously described^17-20^. DNA methylation was measured from whole blood using Illumina HumanMethylation450 (450 K) BeadChip array in 3,927 WHI participants from two studies: the Broad Agency Award 23 (WHI-BAA23), a case-control study of cardiovascular disease, and the Epigenetic Mechanisms of PM-Mediated Cardiovascular Disease Risk (WHI-EMPC), a stratified, random sample (n = 2,200) of participants who were examined between 1993 and 2001. This study includes 3,201 participants (EA, AA, HA) with complete data on DNA methylation, kidney traits and covariates.

#### Ethics

All study participants have provided written consent to participate in genetic studies.

#### DNA Methylation

Methylation data was generated with the Illumina 450K array using baseline visit peripheral blood samples. Preprocessing included removal of probes with detection p values > 0.01 in > 10% of samples and samples with detection p values > 0.01 in 1% of probes. β-values were normalized using beta-mixture quantile (BMIQ) normalization method^21^, and extreme outliers were identified and removed. Batch effect correction was performed using ComBat^22^ or adjusting as a covariate. Cell proportions were estimated using the reference-based Houseman method for whole blood ^23, 24^. Principal components (PCs) were obtained from the genome-wide genotype data available using standard methods ^25^. DNA methylation biomarkers of age acceleration were estimated using probes from the 450K array.

#### Regression analyses

Study-specific modifications to the regression models included adjustment for recruitment region and study samples (BAA vs EMPC).

#### Acknowledgements

The WHI program is funded by the National Heart, Lung, and Blood Institute, National Institutes of Health, U.S. Department of Health and Human Services through contracts HHSN268201600018C, HHSN268201600001C, HHSN268201600002C, HHSN268201600003C, and HHSN268201600004C.” The authors thank the WHI investigators and staff for their dedication, and the study participants for making the program possible. A full listing of WHI investigators can be found at: <http://www.whi.org/researchers/Documents%20%20Write%20a%20Paper/WHI%20Investigator%20Long%20List.pdf> This study was supported by the National Institutes of Health R01- MD012765, R01-DK117445 and R21-HL140385 to NF.

### **JHS**

#### Cohort description

Between 2000 and 2004, JHS recruited 5,306 African American participants from the Jackson, Mississippi, metropolitan tri-county area (Hinds, Madison, and Rankin). JHS (Jackson Heart Study) is a prospective, community-based cohort designed to investigate risk factors for cardiovascular disease among African Americans. A range of measures, including traditional and putative CVD risk factors, health behaviors, detailed demographic, socioeconomic and sociocultural factors, medication use, anthropometry, blood pressure, assessments of kidney function and diabetes, and biochemical analytes, were obtained at the baseline JHS examination and in two subsequent clinic visits (2005-2008 and 2009-2013) ^26-28^. JHS has data for albumin to creatinine ratio from both spot and 24 hour urine collection. Urine albumin was measured by nephalometric immunoassay from Dade-Behring ^26^. Urine creatinine was measured with an enzymatic from Vitros ^26^. eGFR was calculated using the CKD-EPI equation using serum creatinine IDMS calibrated as described in ^29^. Uric acid was measured using the uricase method with the Vitro 5.1 analytical system^30^.

#### Ethics

All participants included in this analysis provided written, informed consent for use of genetic data, and all study protocols conform to the 1975 Declaration of Helsinki guidelines. The study was approved by the Institutional Review Boards of the participating institutions (University of Mississippi Medical Center, Jackson State University and Tougaloo College).

#### DNA Methylation

Illumina Methylation EPIC array data (containing over 850,000 CpG methylation sites) was generated using whole blood samples collected at the JHS baseline exam. Methylation β values (the ratio of intensities between methylated and un-methylated alleles) were normalized with respect to background color intensity using the normal-exponential out-of-band (NOOB) preprocessing method in the R package minfi ^10^. Data on non-duplicate samples passing quality control were then uploaded to Horvath’s online calculator, as described in the main text, to generate the various clock estimates.

#### Regression analyses

Study-specific modifications to the regression models were: 1) inclusion of the first two principal components of genetic data to account for population stratification within the population sample, and 2) the use of a revised 8 CpG MS score, due to use of EPIC data which does not cover all of the 10 CpGs used in the original score; the revised score has r>=0.98 with the original.

Regarding the mortality risk score, a modified form based on 8 CpGs covered by both 450K and EPIC array was used in this study. This adapted MRS showed very strong correlation with the score of 10 CpGs (r>=0.98) in various samples and similarly survival prediction as the 10-CpGs in our validation studies of different types of population. The formula is shown below:

MRscore_8CpG =-0.36909 + cg01612140 *(-1.09957) + cg05575921 *(-1.65446) +

cg08362785 *( 3.12883) + cg10321156 *(-0.22268) +

cg14975410 *(-0.30369) + cg19572487 *(-0.31940) +

cg24704287 *(-3.39726) + cg25983901 *(-1.93238);

#### Acknowledgements

The Jackson Heart Study (JHS) is supported and conducted in collaboration with Jackson State University (HHSN268201800013I), Tougaloo College (HHSN268201800014I), the Mississippi State Department of Health (HHSN268201800015I) and the University of Mississippi Medical Center (HHSN268201800010I, HHSN268201800011I and HHSN268201800012I) contracts from the National Heart, Lung, and Blood Institute (NHLBI) and the National Institute for Minority Health and Health Disparities (NIMHD). The authors also wish to thank the staffs and participants of the JHS.

The views expressed in this manuscript are those of the authors and do not necessarily represent the views of the National Heart, Lung, and Blood Institute; the National Institutes of Health; or the U.S. Department of Health and Human Services. The funders had no role in the design and conduct of the study, in the collection, analysis, and interpretation of the data, and in the preparation, review, or approval of the manuscript.

The project described was supported by the National Center for Advancing Translational Sciences, National Institutes of Health, through Grant KL2TR002490 (LMR). The content is solely the responsibility of the authors and does not necessarily represent the official views of the NIH. LMR was also funded by T32 HL129982.

## **References**

1. Hannum, G, Guinney, J, Zhao, L, Zhang, L, Hughes, G, Sadda, S, et al.: Genome-wide methylation profiles reveal quantitative views of human aging rates. *Molecular cell,* 49**:** 359-367, 2013.

2. Horvath, S: DNA methylation age of human tissues and cell types. *Genome biology,* 14**:** R115, 2013.

3. Levine, ME, Lu, AT, Quach, A, Chen, BH, Assimes, TL, Bandinelli, S, et al.: An epigenetic biomarker of aging for lifespan and healthspan. *Aging,* 10**:** 573-591, 2018.

4. Lu, AT, Quach, A, Wilson, JG, Reiner, AP, Aviv, A, Raj, K, et al.: DNA methylation GrimAge strongly predicts lifespan and healthspan. *Aging,* 11**:** 303-327, 2019.

5. Holle, R, Happich, M, Lowel, H, Wichmann, HE: KORA--a research platform for population based health research. *Gesundheitswesen (Bundesverband der Arzte des Offentlichen Gesundheitsdienstes (Germany)),* 67 Suppl 1**:** S19-25, 2005.

6. Bibikova, M, Barnes, B, Tsan, C, Ho, V, Klotzle, B, Le, JM, et al.: High density DNA methylation array with single CpG site resolution. *Genomics,* 98**:** 288-295, 2011.

7. Zeilinger, S, Kuhnel, B, Klopp, N, Baurecht, H, Kleinschmidt, A, Gieger, C, et al.: Tobacco smoking leads to extensive genome-wide changes in DNA methylation. *PloS one,* 8**:** e63812, 2013.

8. Du, P, Zhang, X, Huang, CC, Jafari, N, Kibbe, WA, Hou, L, et al.: Comparison of Beta-value and M-value methods for quantifying methylation levels by microarray analysis. *BMC bioinformatics,* 11**:** 587, 2010.

9. Lehne, B, Drong, AW, Loh, M, Zhang, W, Scott, WR, Tan, ST, et al.: A coherent approach for analysis of the Illumina HumanMethylation450 BeadChip improves data quality and performance in epigenome-wide association studies. *Genome biology,* 16**:** 37, 2015.

10. Aryee, MJ, Jaffe, AE, Corrada-Bravo, H, Ladd-Acosta, C, Feinberg, AP, Hansen, KD, et al.: Minfi: a flexible and comprehensive Bioconductor package for the analysis of Infinium DNA methylation microarrays. *Bioinformatics (Oxford, England),* 30**:** 1363-1369, 2014.

11. Smyth, GK: limma: Linear Models for Microarray Data. In: *Bioinformatics and Computational Biology Solutions Using R and Bioconductor.*  edited by GENTLEMAN, R., CAREY, V. J., HUBER, W., IRIZARRY, R. A., DUDOIT, S., New York, NY, Springer New York, 2005, pp 397-420.

12. Raum, E, Rothenbacher, D, Low, M, Stegmaier, C, Ziegler, H, Brenner, H: Changes of cardiovascular risk factors and their implications in subsequent birth cohorts of older adults in Germany: a life course approach. *European journal of cardiovascular prevention and rehabilitation : official journal of the European Society of Cardiology, Working Groups on Epidemiology & Prevention and Cardiac Rehabilitation and Exercise Physiology,* 14**:** 809-814, 2007.

13. Zhang, Y, Wilson, R, Heiss, J, Breitling, LP, Saum, K-U, Schöttker, B, et al.: DNA methylation signatures in peripheral blood strongly predict all-cause mortality. *Nature Communications,* 8**:** 14617, 2017.

14. Chen, YA, Lemire, M, Choufani, S, Butcher, DT, Grafodatskaya, D, Zanke, BW, et al.: Discovery of cross-reactive probes and polymorphic CpGs in the Illumina Infinium HumanMethylation450 microarray. *Epigenetics,* 8**:** 203-209, 2013.

15. Mehta, AJ, Zanobetti, A, Bind, MA, Kloog, I, Koutrakis, P, Sparrow, D, et al.: Long-term exposure to ambient fine particulate matter and renal function in older men: The Veterans Administration Normative Aging Study. *Environmental health perspectives,* 124**:** 1353-1360, 2016.

16. Gao, X, Colicino, E, Shen, J, Just, AC, Nwanaji-Enwerem, JC, Wang, C, et al.: Comparative validation of an epigenetic mortality risk score with three aging biomarkers for predicting mortality risks among older adult males. *International journal of epidemiology,* 48**:** 1958-1971, 2019.

17. Design of the Women's Health Initiative clinical trial and observational study. The Women's Health Initiative Study Group. *Control Clin Trials,* 19**:** 61-109, 1998.

18. Anderson, GL, Manson, J, Wallace, R, Lund, B, Hall, D, Davis, S, et al.: Implementation of the Women's Health Initiative study design. *Ann Epidemiol,* 13**:** S5-17, 2003.

19. Howard, BV, Van Horn, L, Hsia, J, Manson, JE, Stefanick, ML, Wassertheil-Smoller, S, et al.: Low-fat dietary pattern and risk of cardiovascular disease: the Women's Health Initiative Randomized Controlled Dietary Modification Trial. *Jama,* 295**:** 655-666, 2006.

20. Jackson, RD, LaCroix, AZ, Gass, M, Wallace, RB, Robbins, J, Lewis, CE, et al.: Calcium plus vitamin D supplementation and the risk of fractures. *N Engl J Med,* 354**:** 669-683, 2006.

21. Teschendorff, AE, Marabita, F, Lechner, M, Bartlett, T, Tegner, J, Gomez-Cabrero, D, et al.: A beta-mixture quantile normalization method for correcting probe design bias in Illumina Infinium 450 k DNA methylation data. *Bioinformatics,* 29**:** 189-196, 2013.

22. Johnson, WE, Li, C, Rabinovic, A: Adjusting batch effects in microarray expression data using empirical Bayes methods. *Biostatistics,* 8**:** 118-127, 2007.

23. Houseman, EA, Accomando, WP, Koestler, DC, Christensen, BC, Marsit, CJ, Nelson, HH, et al.: DNA methylation arrays as surrogate measures of cell mixture distribution. *BMC bioinformatics,* 13**:** 86, 2012.

24. Horvath, S, Levine, AJ: HIV-1 Infection Accelerates Age According to the Epigenetic Clock. *J Infect Dis,* 212**:** 1563-1573, 2015.

25. Price, AL, Patterson, NJ, Plenge, RM, Weinblatt, ME, Shadick, NA, Reich, D: Principal components analysis corrects for stratification in genome-wide association studies. *Nat Genet,* 38**:** 904-909, 2006.

26. Carpenter, MA, Crow, R, Steffes, M, Rock, W, Heilbraun, J, Evans, G, et al.: Laboratory, reading center, and coordinating center data management methods in the Jackson Heart Study. *The American journal of the medical sciences,* 328**:** 131-144, 2004.

27. Taylor, HA, Jr., Wilson, JG, Jones, DW, Sarpong, DF, Srinivasan, A, Garrison, RJ, et al.: Toward resolution of cardiovascular health disparities in African Americans: design and methods of the Jackson Heart Study. *Ethnicity & disease,* 15**:** S6-4-17, 2005.

28. Wilson, JG, Rotimi, CN, Ekunwe, L, Royal, CD, Crump, ME, Wyatt, SB, et al.: Study design for genetic analysis in the Jackson Heart Study. *Ethnicity & disease,* 15**:** S6-30-37, 2005.

29. Wang, W, Young, BA, Fulop, T, de Boer, IH, Boulware, LE, Katz, R, et al.: Effects of serum creatinine calibration on estimated renal function in african americans: the Jackson heart study. *The American journal of the medical sciences,* 349**:** 379-384, 2015.

30. Mwasongwe, SE, Fulop, T, Katz, R, Musani, SK, Sims, M, Correa, A, et al.: Relation of uric acid level to rapid kidney function decline and development of kidney disease: The Jackson Heart Study. *Journal of clinical hypertension (Greenwich, Conn),* 20**:** 775-783, 2018.
